# Development and psychometric properties of the Y-PASS questionnaire to assess correlates of lunchtime and after-school physical activity in children

**DOI:** 10.1186/1471-2458-14-412

**Published:** 2014-04-30

**Authors:** Rebecca M Stanley, Kate Ridley, Timothy S Olds, James Dollman

**Affiliations:** 1 Exercise for Health and Human Performance Group, School of Health Sciences, University of South Australia, Adelaide, South Australia, Australia; 2 Sport, Health and Physical Education Group, School of Education, Flinders University, Adelaide, South Australia, Australia; 3 Health and Use of Time Group, School of Health Sciences, University of South Australia, Adelaide, South Australia, Australia

**Keywords:** Questionnaire development, Physical activity, Psychometric properties, Validity, Reliability, Lunchtime, After-school, Children

## Abstract

**Background:**

To frame interventions, it is useful to understand context- and time-specific correlates of children’s physical activity. To do this, we need accurate assessment of these correlates. There are currently no measures that assess correlates at all levels of the social ecological model, contain items that are specifically worded for the lunchtime and/or after-school time periods, and assess correlates that have been conceptualised and defined by children. The aim of this study was to develop and evaluate the psychometric properties of the lunchtime and after-school Youth Physical Activity Survey for Specific Settings (Y-PASS) questionnaires.

**Methods:**

The Y-PASS questionnaire was administered to 264 South Australian children (146 boys, 118 girls; mean age = 11.7 ± 0.93 years). Factorial structure and internal consistency of the intrapersonal, sociocultural and physical environmental/policy lunchtime and after-school subscales were examined through an exploratory factor analysis. The test-retest reliability of the Y-PASS subscales was assessed over a one-week period on a subsample of children (lunchtime Y-PASS: n = 12 boys, 12 girls, mean age of 11.6 ± 0.8 years; after-school Y-PASS: n = 9 boys, 13 girls; mean age = 11.4 ± 0.9 years).

**Results:**

For the lunchtime Y-PASS, three factors were identified under each of the intrapersonal, sociocultural and physical environmental/policy subscales. For the after-school Y-PASS, six factors were identified in the intrapersonal subscale, four factors in the sociocultural subscale and seven factors in the physical environmental/policy subscale. Following item reduction, all subscales demonstrated acceptable internal consistency (Cronbach alpha = 0.78 – 0.85), except for the lunchtime sociocultural subscale (Cronbach alpha = 0.55). The factors and items demonstrated fair to very high test-retest reliability (ICC = 0.26 – 0.93).

**Conclusion:**

The preliminary reliability and factorial structure evidence suggests the Y-PASS correlate questionnaires are robust tools for measuring correlates of context-specific physical activity in children. The multi-dimensional factor structure provides justification for exploring physical activity correlates from a social ecological perspective and demonstrates the importance of developing items that are context specific. Further development and refinement of the Y-PASS questionnaires is recommended, including a confirmatory factor analysis and exploring the inclusion of additional items.

## Background

A wide range of variables contribute to variance in children’s physical activity. Certain correlates, such as teacher support or access to equipment, may be more strongly associated with physical activity at different times of the day (e.g. school physical activity compared to after-school physical activity) and it is important to identify the relative influence of these correlates in different contexts [1-3]. Correlates are defined as factors that demonstrate reproducible associations or predictive relationships with physical activity [4]. Researchers are calling for additional research into context-specific correlates of children’s physical activity from a social ecological perspective [5-7]. In order to identify the correlates of children’s physical activity occurring at different times of the day, there is a need for accurate assessment of these potential correlates in specific contexts. The accurate assessment and identification of context-specific physical activity correlates through cross-sectional research will assist researchers in better understanding children’s physical activity behaviours and help focus intervention designs to target correlates that are predictive of physical activity and can be modified to bring about positive physical activity behaviour change in specific contexts [5-7].

A number of different correlate measures do exist in the literature, including self-report questionnaires specifically designed to assess location-specific environmental correlates, such as the home, neighbourhood and school environments [8-10] and correlates associated with specific types of physical activity, such as active transport, physical education and leisure activities [11-13]. However, few self-report measures exist that assess correlates of time-specific physical activity in children, such as correlates of before school physical activity or after-school physical activity. Time-specific correlate questionnaires are necessary because they assess the multi-dimensional aspects of physical activity, that is they not only encompass the specific type of activity (e.g. lunchtime play, organised sports and activities, non-organised activities or active transport) but also the location of activity (e.g. school, home, or in the neighbourhood) during the designated time period.

One of the major limitations of current correlate research and measurement is correlate questionnaires are often restricted to a list of ‘global’ correlates or an adult-derived list of correlates that have been predetermined and hypothesised as being relevant to the context under investigation [14-16]. These correlates tend to have limited or poor evidence supporting the relationship with the physical activity context under investigation. For example, social support is a ‘global’ construct but associations may differ depending on the type of activity or the location of the activity [2]. Parental support may be a significant correlate of children’s organised sports but may not be a significant correlate in children’s lunchtime play, whereas teacher support may be associated with children’s lunchtime play but not afterschool play [2]. By including a ‘global’ measure of social support, specific sources of social support will not be identified, which can lead to misconceptions of the primary correlates and possibly biased results. Welk [17] also noted that some adult-derived correlate measurement tools have been reworded and administered to the youth population. There is an assumption that the correlates influencing adults’ physical activity are salient to children’s physical activity [18]. However, adults and children have distinct physical activity patterns and types of behaviours and it cannot be assumed that these measurement tools will capture the range of important correlates specific to children’s activities.

Researchers also tend to use purposely designed correlate questionnaires which have not always been psychometrically tested in the target population or do not cover all domains of the social ecological model [6,19-21]. A social ecological model posits that physical activity behaviour results from multiple influences, including intrapersonal, social and physical environmental factors [22-24]. There are even fewer studies that have used correlate measurement tools where children have been the key informants during development, with some notable exceptions [21,25]. Involving children in the research process can be highly beneficial in gaining further insights into correlates influencing children’s physical activity that may get overlooked when relying on adult-adapted or predetermined correlate questionnaires [26-28].

Two physical activity contexts receiving much attention in recent years are the lunchtime and after-school contexts. These time periods have specifically been identified as critical windows for physical activity promotion during a school day [29,30]. This is because lunchtime and after school are considered discretionary periods, when children are able to make some choices about their participation in physical activity [29]. However, research into the correlates of children’s lunchtime and after-school physical activity is still in its infancy and requires further detailed exploration. There are currently no measures in use that assess potential correlates of lunchtime and after-school physical activity at all levels of the social ecological model, contain questionnaire items that are specifically worded for the lunchtime and/or after-school time periods, and assess potential correlates of lunchtime and after-school physical activity that have been conceptualised and defined by children. In order to contribute to the physical activity correlate body of evidence and advance this field through proposing a context-specific method for assessing correlates, we developed a questionnaire that addresses all of these components. The aim of this study was to develop and evaluate the psychometric properties (i.e. construct validity, internal consistency and stability) of the computer-delivered Youth Physical Activity Survey for Specific Settings (Y-PASS) questionnaire through an exploratory factor analysis and a one-week test-retest study. The Y-PASS questionnaire was designed to assess potential intrapersonal, sociocultural and physical environment/policy correlates of children’s lunchtime and after-school physical activity, which can be used in future cross-sectional studies and inform interventions.

## Methods

### Participants

#### ***Factor analysis***

All students in Grades 5, 6 and 7 (n = 817) from six South Australian schools were invited to take part in this study. The schools included a rural school, a non-Government single-sex girls’ school, a non-Government single-sex boys’ school, a non-Government co-educational school, a high SES Government co-educational school and a low SES Governmental co-educational school. These schools were purposively selected to be reflective of the larger sample by representing both high and low socio-economic areas (SES) according to the School Card Register (SCR). The SCR is an indicator of SES at the school level based on the percentage of students in a school whose families receive government support to meet the costs of school attendance (SCR cut-off for low SES = 31.8%; 50^th^ percentile). Informed consent from a parent or guardian and assent was obtained for 275 participants, giving a response rate of 34%. Of the 275 participants, 264 participants (146 boys, 118 girls; mean age = 11.7 ± 0.93 years) completed either the lunchtime, after-school or both questionnaires, with 189 participants completing the lunchtime questionnaire and 240 participants completing the after-school questionnaire. Eleven students who provided consent did not complete a questionnaire because they were absent on the day of data collection.

To determine whether the sample size estimation was appropriate to establish a factorial structure with minimal sampling error and sufficient stability in the current study, a post hoc judgement was made according to Bartlett’s test of sphericity [31] and the recommended “rules of thumb”. Based on the Hutcheson and Sofroniou [32] rule of a total of 150 to 300 participants, and the significance rule of 51 more cases than the number of variables stated by Lawley and Maxwell [33], the sample size for this study was appropriate to conduct the factor analysis, with the lunchtime questionnaire containing 50 potential variables with a sample size of 189 and the after-school questionnaire containing 109 variables with a sample size of 240. Furthermore, the Bartlett’s test of sphericity produced a significant value, suggesting that there was a factor structure inherent in the data and therefore the sample size was sufficient to reliably estimate correlation coefficients.

#### ***Test-retest reliability***

An independent subsample of children was recruited to explore the test-retest reliability of the Y-PASS questionnaires. Seventy-two participants across Grades 5, 6 and 7 (24 students from each year level) were invited from two primary schools. Informed consent and assent were obtained from 47 parents and participating children, respectively. One child was absent on the days of data collection, resulting in 46 children completing the questionnaires at the two time periods (lunchtime Y-PASS: n = 12 boys, 12 girls, mean age of 11.6 ± 0.8 years; after-school Y-PASS: n = 9 boys, 13 girls; mean age = 11.4 ± 0.9 years). This sample size is comparable with other test-retest reliability studies [8,34,35]. Complete datasets were obtained from 64% of all children approached for this study.

Both studies were approved by the University of South Australia Human Research Ethics Committee, Department of Education and Children Services (DECS), the South Australian Commission for Catholic Schools (SACCS) and from the relevant school authorities.

### Questionnaire development and pilot testing

The development of the Y-PASS questionnaires followed similar processes identified by Frazer and Lawley [36], DeVellis [37] and Streiner and Norman [38] and was based on the social ecological theoretical framework (i.e. intrapersonal, sociocultural environment and physical environment/policy domains) [24]. The content for the original pool of Y-PASS items was informed by the evidence generated from a comprehensive systematic review of the quantitative correlate literature [39] and focus groups conducted with 54 South Australian children aged 10 to 14 years exploring perceptions of influences of lunchtime and after-school physical activity [40,41]. A total of 11 focus groups were conducted and facilitated by the first author (RMS) and supported by a trained research assistant. To assist in the development of questionnaire items and to avoid duplication of existing items, existing correlate questionnaires with acceptable psychometric properties were reviewed and appropriate items were selected and modified to be context-specific, such as “I think I can be physically active even if my friends don’t want to” [42] was modified to “I am confident that I can still be active at lunchtime even if my friends don’t want to”. Items were purposely developed for correlates not addressed by existing questionnaire (see Additional file 1). Using a similar approach adopted and reported in a number of questionnaire development studies [42-45], items thought to reflect intrapersonal, sociocultural and physical environmental/policy domains were divided into corresponding subscales. The division of items was guided by the literature [6,17,24]. The research team met to discuss the wording of each item and the response format. A 5-point Likert scale (i.e. disagree a lot, disagree a little, neither disagree nor agree, agree a little and agree a lot) was developed for the Y-PASS questionnaires as this is deemed appropriate for children [46] and has been used and tested in other questionnaires administered to children of similar age to participants in this study [3,42,47].

A preliminary list of 55 lunchtime-specific and 128 after-school-specific correlate items reflecting different aspects of the social ecological model was reviewed by a panel of ten experts in children’s physical activity, questionnaire development and correlates of physical activity to assess content and face validity. The expert panel represented three different countries (United States, New Zealand and Australia) and have an average *h*-index of 17.7 (*h*-index range = 2 – 43; an index to evaluate productivity and impact of published work of the researcher [48]). The panel were asked to comment on the terminology used, response format, order of the items, length of the questionnaire and the readability. In addition, they were given the opportunity to identify additional items that were not considered in the questionnaires and provide further comments that may improve the content, design and usability of the questionnaires. Recommendations from the expert panel resulted in modification of terminology and wording of items to improve readability and comprehension [38,49] and the removal of five lunchtime and 19 after-school items. The following are examples of items reworded based on the feedback from the expert panel: “There is always a teacher who supervises us during lunchtime” was modified to “There is always a teacher who is on yard duty during lunchtime”, and “I like to walk and talk at lunchtime” was modified to “I like to walk around at lunchtime”.

The second draft of the Y-PASS questionnaires underwent pilot testing with a subsample of children aged 10–14 years in a classroom setting (n = 21 [8 girls, 13 boys], mean age = 11.4 [±0.80] years). Pilot testing provided an opportunity to evaluate and refine aspects of the questionnaires, such as the wording, item order, usability and aesthetics, design and layout features. In particular, the pilot tests were used to assess whether the items were posed from the child’s perspective and not an adult-centric perspective, which tends to be one of the issues with questionnaires developed by adults for children [16]. An example of an item that was reworded based on the pilot testing was, “Our school play area has painted lines on the ground to help me be active at lunchtime”. Examples of painted lines were included into the item to ensure appropriate interpretability (“Our school play area has painted lines on the ground (e.g. hopscotch and 4-square) to help me be active at lunchtime”). As the Y-PASS questionnaires are computer-delivered, it was also important to test the technological aspects of the questionnaires [50]. Based on the overall feedback from the pilot test, modifications were made to the visual aspects of the questionnaires, such as the inclusion of more pictures, using an alternative font, increasing the space between items and changing the colours of headings.

The version of Y-PASS questionnaire used in the current study contained 50 lunchtime items and 109 after-school items. The Flesch-Kincaid Grade level and Flesch Reading Ease scores [51] for the lunchtime questionnaire were 4.96 and 78.90, and 5.76 and 74.11 for the after-school questionnaire, respectively. These scores suggest that the questionnaires are easily understood by Grade 6 and readable by Grade 5. The disparity between the Reading Ease scores and the age group in this study is acknowledged as a limitation of the questionnaires. The items for each subscale and the sources of the items are presented in Additional file 1.

### Data collection

Data collection for the factor analysis occurred between October and November 2010 and between May and June 2011 for the test-retest study. Both studies followed a similar protocol. The computer-delivered lunchtime and after-school Y-PASS questionnaires were administered to participants in a school computer lab during class time. Copies of these questionnaires are presented in Additional file 2. The principal administrator (RMS) read out a standardized script, guiding the participants through the initial pages of the questionnaires. Children participating in the test-retest study completed the questionnaires on two occasions at the same time of day, one week apart. Research assistants were available to answer any questions or assist participants to complete the questionnaires. Children took on average 14.01 (±2.9) minutes to complete the lunchtime Y-PASS questionnaire (range = 11.42 – 16.55 minutes), while the after-school Y-PASS questionnaire took on average 24.50 (±3.9) minutes to complete (range = 19.15 – 28.12 minutes). This variation in completion time is acceptable with child participants [16].

### Data analysis

An exploratory factor analysis was used to determine the factor structure of each Y-PASS subscale. There has been considerable criticism over the individual use of factor extraction methods. Kaiser’s criterion method (i.e. eigenvalues of >1) [52] and the Cattel’s scree test [53] can greatly under- and over-estimate the number of factors to be retained, depending on the number of items. Therefore, Horn’s Parallel Analysis was used to ascertain initial estimate of the number of factors to extract as this has been shown to be the most accurate and an optimal technique for determining the number of factors to be retained [54]. Principal Component extraction with Varimax rotation was conducted for each subscale separately to identify and retain interpretable factors. As the purpose for conducting the exploratory factor analysis was to determine the underlying structure and identify uncorrelated factors, Varimax rotation was deemed most appropriate [55]. Items were assigned to a factor if pattern coefficient loadings were above ±0.45 and did not cross-load with a significant loading of ±0.3 or above onto any other factor [55]. If items did cross-load, the decision of placement with a factor was based on conceptual reasoning [55,56] or these were removed altogether. Items that did not load onto a factor were still retained but removed from the model and treated as individual correlate items rather than correlate factors. Cronbach alpha was tested to determine the internal consistency of the revised subscales and for each individual factor identified. A Cronbach alpha of greater than or equal to 0.6 was interpreted as acceptable [8,57]. Factors were refined by identifying redundant items that did not contribute to the overall internal consistency [56]. In addition, the level of consistency among the items (i.e. the correlation of each item with the total factor score and the proportion of variance in a given item that is shared with the other items) were also reviewed to assist in the decision to remove an item from a factor [56]. To test the stability of questionnaires, intraclass correlation coefficients (ICC) were calculated. The guidelines suggested by Landis and Koch [58] were used to interpret the test-retest coefficients: 0 – 0.2 (poor), 0.21 – 0.4 (fair), 0.41 – 0.6 (moderate), 0.61 – 0.8 (substantial) and 0.81 – 1.0 (almost perfect) [59]. All analyses were conducted using Statistical Package for the Social Sciences (SPSS) Version 17.0 (SPSS, Chicago, IL) software and STATA Version 11 (StataCorp LP, Texas, USA) software was used to execute the Parallel Analyses.

## Results

### Evaluation of the appropriateness of factor analysis

Data were initially reviewed for appropriateness of factor analysis. Examination of the correlation matrix of each subscale revealed that there were correlation coefficients greater than 0.3, suggesting some clustering of items was expected and exploratory factor analysis deemed appropriate [60]. Subscale Cronbach alphas ranged from 0.61 to 0.88, which is between the recommended values of 0.60-0.90 [57]. Bartlett’s test of sphericity reached statistical significance (p < 0.000) [60] and KMO values for the subscales ranged from 0.65 to 0.84, which exceeds the minimum recommended value of 0.60 [61] (see Table 1). These values indicate the appropriateness of exploratory factor analysis for the lunchtime and after-school subscales.

**Table 1:** Cronbach alpha, Kaiser-Meyer-Olkin (KMO) measure of sampling adequacy and Bartlett’s test of sphericity for the intrapersonal, sociocultural and physical environmental/policy subscales of the Y-PASS questionnaires

**Subscale**	**Cronbach alpha**	**KMO**	**Bartlett’s test**
Lunchtime Y-PASS			
Intrapersonal	0.84	0.84	1539 p < 0.000
Sociocultural	0.61	0.65	227 p < 0.000
Physical environmental/policy	0.70	0.75	438 p < 0.000
After-school Y-PASS			
Intrapersonal	0.88	0.84	4356 p < 0.000
Sociocultural	0.77	0.74	1765 p < 0.000
Physical environmental/policy	0.81	0.70	2070 p < 0.000

### Lunchtime Y-PASS

#### ***Intrapersonal subscale***

The final number of factors for the intrapersonal subscale was three, accounting for 48.5% of the explained variance (see Table 2). Following the removal of redundant items, the internal consistency for this subscale was 0.85. Factor one was labelled “Barrier self-efficacy” and had significant loadings from seven items (Cronbach alpha = 0.80; ICC = 0.84). Six items loaded significantly onto the second factor, interpreted as “Perceived self-efficacy” (Cronbach alpha = 0.78; ICC = 0.73). Factor three, labelled as “Behavioural attitude/belief”, had significant loadings from five items (Cronbach alpha = 0.78; ICC = 0.73). Six items either did not load onto any factor or cross-loaded and therefore, remained as individual correlate items (ICC ranging from 0.33 – 0.67).

**Table 2:** Factor analysis after rotation (sorted by size) for the lunchtime intrapersonal subscale, rotated component matrix (n = 189)

**Item**	**Factor**		
	**Barrier self-efficacy**	**Perceived self-efficacy**	**Behavioural attitude/belief**
I am confident that I can find other kids to be active with at lunchtime even if my friends don’t want to.	0.78		
I am confident that I can be active at lunchtime even if the space in the playground/oval is limited.	0.73		
I am confident that I can ask a teacher to get me equipment to play with at lunchtime.	0.70		
I am confident that I can still be active at lunchtime even if my friends don’t want to.	0.64		
I am confident that I can still be active in the school yard even if it is very hot or raining.	0.63		
I am confident that I can ask my friends to be active with me during lunchtime.	0.61		
I am confident that I can still be active at lunchtime even if there are bullies in the school yard.	0.53		
I prefer to watch other kids rather than play active games at lunchtime.		0.74	
I am not good at being active at lunchtime.		0.72	
I prefer to sit rather than be active at lunchtime.		0.66	
There is nothing to do at lunchtime.		0.60	
It is fun to be active at lunchtime.		0.57	
I have the skills I need to be active at lunchtime.		0.52	
I am active at lunchtime so I can hang out with my friends.			0.73
I play certain games at lunchtime because I think I am good at them.			0.70
I am active at lunchtime because it makes me popular with the other children.			0.66
It is 'cool' to be active at lunchtime.			0.57
I play certain games at lunchtime because I want to get extra practice.			0.56
**Eigenvalue**	**3.46**	**3.44**	**3.29**
**% variance explained**	**16.5**	**16.4**	**15.7**
**Cronbach alpha**^**a**^	**0.80**	**0.78**	**0.78**
**Total % variance explained**			**48.5**
**Total subscale Cronbach alpha**^**a**^			**0.85**

*Notes*: Extraction method: Principal Component Analysis; Rotation Method: Varimax with Kaiser Normalisation; ^a^An acceptable Cronbach alpha is ≥0.6 [8,57].

#### ***Sociocultural subscale***

All items measuring a sociocultural construct loaded significantly onto one of three interpretable factors (i.e. “Peer influence”, “Teacher influence” or “Social Barriers”). However, the item “Teachers encourage us to be active at lunchtime” was removed as it did not contribute to the overall Cronbach alpha. The internal consistency of this subscale was 0.55 and accounted for 55.4% of the total variance of the factor solution. Test-retest reliability ranged from 0.57 – 0.70 (see Table 3).

**Table 3:** Factor analysis after rotation (sorted by size) for the lunchtime sociocultural subscale, rotated component matrix (n = 189)

**Item**	**Factor**		
	**Peer influence**	**Teacher influence**	**Social barriers**
I teach other children how to play active games at lunchtime.	0.82		
My friends teach me how to play active games at lunchtime.	0.78		
I have friends who I am active with at lunchtime.	0.61		
Teachers help us with the active games we play at lunchtime.		0.83	
Teachers play with us at lunchtime.		0.80	
There is always a teacher who is on yard duty during lunchtime.			0.70
My friends would rather sit and talk at lunchtime.			0.61
Bullying stops me from being active in the school yard at lunchtime.			0.58
**Eigenvalue**	**1.85**	**1.68**	**1.46**
**% variance explained**	**20.5**	**18.7**	**16.2**
**Cronbach alpha**^**a**^	**0.64**	**0.64**	**0.32**
**Total % variance explained**			**55.4**
**Total subscale Cronbach alpha**^**a**^			**0.55**

*Notes*: Extraction method: Principal Component Analysis; Rotation Method: Varimax with Kaiser Normalisation; ^a^An acceptable Cronbach alpha is ≥0.6 [8,57].

#### ***Physical environmental/policy subscale***

The three-factor solution for physical environmental/policy subscale had an internal consistency of 0.74 and accounted for 52.8% of the variance of the factor solution (see Table 4). The first factor, “Access to facilities/equipment”, had three items loading significantly (Cronbach alpha = 0.61; ICC = 0.71). “Physical environmental/policy barriers” was identified as the second factor and consisted of three items (Cronbach alpha = 0.55; ICC = 0.66). Three items loaded significantly onto the “Access to space” factor (Cronbach alpha = 0.50; ICC = 0.60). Three items were removed from the model but remained as correlate items because they either cross-loaded onto multiple factors or did not load onto any factor (ICC = 0.46 – 0.75). The item, “Our school has areas that suit the games I want to play at lunchtime”, was discarded due to poor test-retest reliability (ICC = 0.08).

**Table 4:** Factor analysis after rotation (sorted by size) for the lunchtime physical environmental/policy subscale, rotated component matrix (n = 189)

**Item**	**Factor**		
	**Access to facilities/equipment**	**Physical environmental/policy barriers**	**Access to space**
There are lots of shaded areas where I can be active even if it is really hot.	0.70		
There are indoor spaces where I can be active if it is raining.	0.70		
There is enough equipment available for me to play with at lunchtime.	0.65		
It is hard to be active in our school uniform at lunchtime.		0.67	
The oval is too dry and hard to play on.		0.67	
Some school rules keep me from doing the activities I like at lunchtime.		0.58	
There is enough space in the school yard for me to be active at lunchtime.			0.84
There is enough grass in the school yard to be active at lunchtime.			0.58
There are too many kids in the playground for me to be active at lunchtime.			0.53
**Eigenvalue**	**1.92**	**1.78**	**1.58**
**% variance explained**	**19.2**	**17.8**	**15.8**
**Cronbach alpha**^**a**^	**0.61**	**0.55**	**0.50**
**Total % variance explained**			**52.8**
**Total subscale Cronbach alpha**^**a**^			**0.74**

*Notes:* Extraction method: Principal Component Analysis; Rotation Method: Varimax with Kaiser Normalisation; ^a^An acceptable Cronbach alpha is ≥0.6 [8,57].

### After-school Y-PASS

#### ***Intrapersonal subscale***

The items in the intrapersonal subscale loaded significantly onto six factors, as identified by the Parallel Analysis. The overall subscale explained 54.1% of the variance and had an internal consistency of 0.85 (see Table 5). Factor one and factor two both explored “Behavioural attitudes/beliefs” with factor one specifically targeting organised sports and activities (seven items, Cronbach alpha = 0.77; ICC = 0.85) and factor two targeting non-organised activities (six items, Cronbach alpha = 0.82; ICC = 0.93). Six items loaded onto the third factor, which explored aspects of “Barrier self-efficacy” (Cronbach alpha = 0.80; ICC = 0.73). Factor four (three items) was labeled “Support seeking/social norm” (Cronbach alpha = 0.70; ICC = 0.58) and factor five (“Perceived competence”) had two items (Cronbach alpha = 0.82; ICC = 0.73). The final factor identified in the intrapersonal subscale was interpreted as a “Perceived barriers” factor, consisting of three items (Cronbach alpha = 0.43; ICC = 0.62). A number of items (n = 16) did not load onto any factor or cross-loaded onto multiple factors and these were removed from the model but remained as correlate items.

**Table 5:** Factor analysis after rotation (sorted by size) for the after-school intrapersonal subscale, rotated component matrix (n = 240)

**Item**	**Factor**					
	**Behavioural attitudes/beliefs (organised sports/activities)**	**Behavioural attitudes/beliefs (non-organised activities)**	**Barrier self-efficacy**	**Support seeking/social norm**	**Perceived competence**	**Perceived barriers**
I don't do an organised sport or activity after school because other kids are better than me.	0.67					
I don't feel like doing an organised sport or activity after school.	0.66					
I enjoy being part of an organised sport or activity team.	0.65					
I don't enjoy doing an organised sport or activity after school.	0.62					
It is not worth doing an organised sport or activity after school because I am not good at it.	0.59					
I am not active after school because I am scared that I will get injured.	0.52					
I prefer to watch other kids rather than do organised sports and activities after school.	0.49					
I prefer to be active after school instead of watching TV or playing electronic games.		0.68				
Being active after school is the thing I like to do best.		0.66				
I don't feel like playing actively at home or in the neighbourhood after school.		0.60				
Being active after school makes me feel good.		0.55				
I am too tired to be active after school.		0.54				
It is fun being active after school.		0.53				
I am confident that I can ask my parent or another adult to take me somewhere I can play actively after school.			0.81			
I am confident that I can ask my parent or another adult to take me to an organised sport or activity after school.			0.69			
I am confident that I can be active after school on most days even if I have to stay at home.			0.65			
I am confident that I can be active after school on most days.			0.63			
I am confident that I can ask friends to be active with me after school on most days.			0.62			
I am confident that I can be active after school on most days no matter how busy I am.			0.57			
I play in the neighbourhood after school because I get to hang out with my friends.				0.76		
I walk or ride to and from places after school because I get to hang out with my friends.				0.74		
I play in the neighbourhood after school because I get to meet new people.				0.70		
I play active games after school because I think I am good at them.					0.86	
I do an organised sport or activity after school because I think I am good at it.					0.80	
I prefer to do homework rather than be active after school.						0.63
I am scared of strangers in my neighbourhood after school.						0.62
I am scared of dangerous animals in my yard, such as snakes, lizards, dogs or magpies.						0.59
**Eigenvalue**	**3.49**	**3.34**	**3.15**	**2.46**	**2.09**	**1.69**
**% variance explained**	**11.6**	**11.1**	**10.5**	**8.2**	**7.0**	**5.6**
**Cronbach alpha**^**a**^	**0.77**	**0.82**	**0.80**	**0.70**	**0.82**	**0.43**
**Total % variance explained**						**54.1**
**Total subscale Cronbach alpha**^**a**^						**0.85**

*Notes:* Extraction method: Principal Component Analysis; Rotation Method: Varimax with Kaiser Normalisation; ^a^An acceptable Cronbach alpha is ≥0.6 [8,57].

#### ***Sociocultural subscale***

Four factors emerged in the sociocultural subscale, explaining 49.0% of the variance with an internal consistency of 0.75 (see Table 6). Seven items loaded onto the “Social support” factor (Cronbach alpha = 0.78; ICC = 0.91) and four items loaded onto the “Parental barriers” factor (Cronbach alpha = 0.63; ICC = 0.55). The third factor was labeled “License to be active” (Cronbach alpha = 0.75; ICC = 0.85). The final factor was interpreted as “Parental rules” and consisted of two items (Cronbach alpha = 0.56; ICC = 0.77). Eight items did not load onto any of these factors or cross-loaded onto multiple factors, and as a result, were removed from the model but remained as individual correlate items (ICC = 0.31 – 0.75).

**Table 6:** Factor analysis after rotation (sorted by size) for the after-school sociocultural subscale, rotated component matrix (n = 240)

**Item**	**Factor**			
	**Social support**	**Parental barriers**	**License to be active**	**Parental rules**
My parents help me practise sport after school.	0.77			
My parents play actively with me after school.	0.73			
My family tell me I am doing well at my after-school organised sport or activity.	0.60			
My family always watch me do an organised sport or activity after school.	0.58			
My parents encourage me to play outside after school.	0.56			
My parents encourage me to do an organised sport or activity after school.	0.53			
I do an organised sport or activity with friends after school.	0.49			
I don’t do an organised sport or activity after school because my parents work late.		0.75		
I am not allowed to do an organised sport or activity after school because my parents are scared that I might get hurt.		0.73		
My parents won’t let me do an organised sport or activity because I am already doing too many other activities.		0.61		
My parents are not home after school to supervise my play.		0.47		
My parents won’t let me ride, walk, skate or scooter to and from places after school.			0.78	
I walk, ride, skate or scooter to and from places with friends after school.			0.73	
My parents think it is safe for me to be active in the neighbourhood after school.			0.72	
I play with friends in the neighbourhood after school.			0.58	
If I am going out after school, I always have to be back by a certain time.				0.76
I always have to tell my parents where I am when I go out after school.				0.75
**Eigenvalue**	**3.22**	**2.47**	**2.46**	**1.66**
**% variance explained**	**16.1**	**12.4**	**12.3**	**8.3**
**Cronbach alpha**^**a**^	**0.78**	**0.63**	**0.75**	**0.56**
**Total % variance explained**				**49.0**
**Total subscale Cronbach alpha**^**a**^				**0.75**

*Notes:* Extraction method: Principal Component Analysis; Rotation Method: Varimax with Kaiser Normalisation; ^a^An acceptable Cronbach alpha is ≥0.6 [8,57].

#### ***Physical environmental/policy subscale***

For the physical environmental/policy subscale, a seven factor solution was identified, explaining 52.6% of the variance (see Table 7). The internal consistency of this subscale was 0.78. “Weather” (six items, Cronbach alpha = 0.75; ICC = 0.69), “Access to facilities/equipment” (seven items, Cronbach alpha = 0.69; ICC = 0.80) and “Safety” (four items, Cronbach alpha = 0.63; ICC = 0.75) were identified in the factor structure. Factor four was labeled “Access to space” (Cronbach alpha = 0.60; ICC = 0.75), while the fifth factor was labeled “Time commitments” (Cronbach alpha = 0.61; ICC = 0.66). Three items loaded significantly onto the “Financial barriers” factor (Cronbach alpha = 0.64; ICC = 0.60). The final factor of the physical environmental/policy subscale was deciphered as a “School bag” factor and contained two items (Cronbach alpha = 0.54; ICC = 0.53). These items related to the school bag being a barrier to engaging in active transport. Five items were removed from the model due to factor loadings of less than 0.45 or cross-loading onto multiple factors. However, these items did remain in the questionnaire as individual correlate items (ICC = 0.27 – 0.80).

**Table 7:** Factor analysis after rotation (sorted by size) for the after-school physical environmental/policy subscale, rotated component matrix (n = 240)

**Item**	**Factor**						
	**Weather**	**Access to facilities/equipment**	**Safety**	**Access to space**	**Time commitments**	**Financial barriers**	**School bag**
When it is too hot, it stops me from playing actively after school.	0.69						
When it is raining, it stops me from playing actively after school.	0.68						
When it is raining, it stops me from walking, riding, skating or riding a scooter to and from places after school.	0.68						
When it is too hot, it stops me from walking, riding, skating or riding a scooter to and from places after school.	0.67						
When it is too hot, it stops me from doing an organised sport or activity after school.	0.57						
When it is raining, it stops me from doing an organised sport or activity after school.	0.55						
It is easy to get to an organised sport or activity after school.		0.67					
There are playgrounds or parks near my house where I can be active after school.		0.59					
I don’t have to travel far to play with my friends after school.		0.58					
There are sport or recreation centres that I can go to after school.		0.55					
I have the right equipment to do my chosen organised sport or activity after school.		0.51					
I live too far away to walk, ride, skate or scooter to and from places after school.		0.48					
I have the right equipment (e.g. a bike lock, helmet or bike) to ride a bike after school.		0.46					
The roads are safe in my neighbourhood after school.			0.74				
There is heavy traffic in the streets where I live.			0.71				
There are dangerous objects in my yard, such as rusty scrap metal.			0.59				
It is safe to play actively in my yard after school.			0.55				
My yard is too small for me to be active after school.				0.79			
I play actively in my yard after school because I have a lot of lawn.				0.72			
There is somewhere at home where I can play actively after school.				0.60			
Homework stops me from doing an organised sport or activity after school.					0.77		
Homework stops me from playing actively at home or in the neighbourhood after school.					0.70		
We do not have enough cars to drive to and from places where I can be active after school.						0.78	
Petrol costs too much to drive to and from places where I can be active after school.						0.76	
It costs too much money to do an organised sport or activity after school.						0.64	
My school bag(s) is too heavy for me to walk, ride, skate or scooter home after school.							0.60
I don’t walk, ride, skate or scooter home from school when I have too many bags to carry.							0.59
**Eigenvalue**	**2.85**	**2.74**	**2.08**	**2.02**	**2.02**	**1.98**	**0.56**
**% variance explained**	**9.8**	**9.5**	**7.2**	**7.0**	**7.0**	**6.8**	**5.4**
**Cronbach alpha**^**a**^	**0.75**	**0.69**	**0.63**	**0.60**	**0.61**	**0.64**	**0.54**
**Total % variance explained**							**52.6**
**Total subscale Cronbach alpha**^**a**^							**0.78**

*Notes:* Extraction method: Principal Component Analysis; Rotation Method: Varimax with Kaiser Normalisation; ^a^ An acceptable Cronbach alpha is ≥0.6 [8,57].

## Discussion

Valid and reliable measures of potential context-specific physical activity correlates for use with children are lacking but are fundamental for exploring and understanding the factors that could be targeted in interventions to promote physical activity. This study presented preliminary evidence of the construct validity, internal consistency and test-retest reliability of six new context-specific physical activity correlate subscales: lunchtime intrapersonal subscale, lunchtime sociocultural subscale, lunchtime physical environment/policy subscale, after-school intrapersonal subscale, after-school sociocultural subscale and the after-school physical environmental/policy subscale.

Results from the exploratory factor analysis demonstrated the existence of a factor structure among the Y-PASS questionnaire items. For the lunchtime subscales, three factors were identified under each of the intrapersonal, sociocultural and physical environmental/policy subscales. For the after-school subscales, six factors were identified in the intrapersonal subscale, four factors in the sociocultural subscale and seven factors in the physical environmental/policy subscale. Following item reduction, all subscales, except the lunchtime sociocultural subscale (Cronbach alpha = 0.55), demonstrated acceptable internal consistency (defined as a Cronbach alpha of ≥0.6 [8,57]). Within the subscales, however, there were some factors that demonstrated moderate internal consistency. For example, the “Parental rules” factor in the after-school sociocultural subscale and the “Access to space” in the lunchtime physical environment/policy subscale had Cronbach alpha values of 0.56 and 0.50, respectively. This may be due to only two and three items loading onto these factors. Of particular note was the low Cronbach alpha for the “Social barriers” factor in the lunchtime sociocultural subscale (Cronbach alpha = 0.32) and “Perceived barriers” of the after-school intrapersonal subscale (Cronbach alpha = 0.43), with only three items loading onto each of these factors. According to Pallant [60], the mean inter-item correlation may be a more appropriate statistic to determine internal consistency of factors with a small number of items. The “Social barriers” and “Perceived barriers” factors have mean inter-item correlations of 0.15 and 0.20, respectively, which are in the acceptable range of 0.10 to 0.50 [62]. In light of this knowledge, future studies using the Y-PASS questionnaires could consider repeating a factor analysis with additional items for the factors demonstrating low or moderate Cronbach alpha values, and if the factorial structure is maintained, testing whether this raises the alpha values. Alternatively, analyses could be conducted with individual correlate items, rather than amalgamating items into factors, to explore relationships with physical activity outcomes.

The social ecological model posits that physical activity is subjected to multiple influences [24], which is reflected in the multi-dimensional factorial structure identified in this study. Similar findings have been found in a number of other studies reporting the factorial structure of newly developed correlate measures [21,42-45]. The inclusion of multiple factors under each subscale increases the level of measurement specificity and may contribute to explaining more variance in children’s physical activity behaviour.

The factors identified under the lunchtime and after-school intrapersonal subscales measure different facets of the intrapersonal domain, such as self-efficacy and behavioural attitudes/beliefs. In a study with children, Ommundsen et al. [21] also explored different dimensions of the intrapersonal domain and identified a two factor solution (i.e. enjoyment and perceived competence). There have been a number of other studies conducted with children that have explored the factor structure of an intrapersonal subscale but have only focused on one aspect of this domain. For example, Pirasteh et al. [44] only explored self-efficacy and found that items only loaded onto one factor. This limits the depth of exploration into the internal psychological processes that influence children’s engagement in physical activity. Saunders et al. [42], on the other hand, also explored the factorial structure of the self-efficacy domain but identified a three factor solution (i.e. support seeking, barriers, positive alternatives), which explores a deeper level of specificity to this factor. In order to obtain an in-depth understanding of why children are active or not active, different dimensions of children’s intrapersonal domain should be explored instead of focusing on single dimensions.

In this study, multiple factors measuring different aspects of the sociocultural domain were identified, including aspects of peer, teacher and parental influences. This is supported by findings from Ommundsen et al. [21] and Pirasteh et al. [44] who identified similar factors. On the other hand, Saunders et al. [42] and Robertson-Wilson et al. [10] only identified a single factor structure for the social subscale, with peer, family and teacher influence collapsed into one factor. The disadvantage of a single factor solution is the inability to assess the specific influence of peers, family or teachers on physical activity separately and in different contexts. Evidence has shown that children do relate differently to different social groups in different settings [2,3,43], suggesting that a multi-dimensional factor structure may be more appropriate, particularly when exploring contextual correlates of physical activity.

The factor structure of a physical environmental/policy subscale has not been explored in detail in the literature [47]. Robertson-Wilson et al. [10] identified a single factor solution for the physical environment domain, which lumped together items assessing the condition of space and equipment, size of space, access to space, access to equipment and access to physical education classes and organised activities. The issue with collapsing multiple aspects of a domain into one factor is a loss of specificity and an inability to identify the specific environmental aspect that influences physical activity behaviour. In comparison, Hume et al. [8] and McMinn et al. [45] explored the psychometric properties of a physical environment scale and concluded that multiple dimensions of the physical environment (e.g. safety, aesthetics, access to facilities, availability of equipment) should be explored when investigating the factors that influence physical activity. The evidence from these studies, along with the multi-factor solution identified from this current exploratory factor analysis, provides support for the use of a range of items that cover different potential physical environmental factors so that all underlying factors are captured.

While the majority of correlate items and factors demonstrated fair to excellent test-retest reliability over a one-week period, there were a few exceptions. There are several possible reasons for poor stability of some items and factors in questionnaires over time. Difference in responses over time may reflect true changes in an individual’s behaviour or subjective responses, such as opinions and feelings, which may vary substantially from week to week [63,64]. These are perhaps affected by the experiences on the day of data collection. For example, the item “Bullying stops me from being active after school” may have been important for a participant during one week but not the other, or the participant’s perception of the item “My friends encourage me to be active after school” may have been influenced by the presence of peers during after-school playtime. It has also been suggested that characteristics of the sample, maturity level, changes in the respondent’s emotional state, differences in the testing situation, recollection of previous answers, and difficulty with understanding items and the construct being tested can all affect how participants respond to items on two different occasions [56]. While some of these factors cannot be controlled, such as the characteristics of the sample and maturity level, a number of strategies were implemented during the data collection procedures to minimise the impact of these factors on the final results, such as keeping each testing administration as consistent as possible by using the same computers, having the same research assistants available and administering the questionnaires at the same time of day.

The test-retest reliability findings for the Y-PASS questionnaires are consistent with other questionnaires measuring conceptually similar correlates. For example, test-retest reliability for self-efficacy ranged from 0.73-0.84 in the current study, which is similar to the test-retest reliability of a self-efficacy variable used in questionnaires reported by Pirasteh et al. [44] (Pearson correlation coefficient = 0.68), Pate et al. [65] (r = 0.76) and Trost et al. [66] (r = 0.76 – 0.82) in similar aged samples. Stevens et al. [67] reported a lower test-retest reliability value for self-efficacy (r = 0.58). However, this was over a 3–6 week test-retest period, compared to the other studies which ranged between 1–2 weeks.

The average test-retest reliability across the lunchtime Y-PASS and after-school Y-PASS physical environmental/policy subscales (ICC = 0.61 and 0.62, respectively) was lower than the test-retest reliability reported by Robertson-Wilson et al. [10] (r = 0.78) but higher than the school environment variable reported by Wong et al. [64] (kappa = 0.42) and the physical environment factor reported by Pirasteh et al. [44] (r = 0.38). This is an unusual finding because physical environmental factors should be relatively stable over time. Indeed, individual environmental factors are more likely to change from week to week (e.g. changes in the weight of school bags and dog poo on the lawn at home), which has been supported by the lower test-retest reliability coefficients of the Y-PASS items. However, it is unlikely that school and neighbourhood environments dramatically change in one week (e.g. facilities at school, school policies, traffic lights and crossings in the neighbourhood). This is not supported by the current findings of this study. For example, the items relating to traffic lights and crossings in the neighbourhood and facilities at school had test-retest reliabilities of 0.47 and 0.46, respectively. Hume et al. [8] and Erwin [34] also reported weak stability of similar physical environmental factors. It is important to note that the Y-PASS questionnaire items, as well as the questionnaires used in the studies conducted by Hume et al. [8] and Erwin [34], assessed children’s perceptions and while the actual environmental factors may not change, it is children’s perceptions of these factors that may change across a week, resulting in lower test-retest reliability. It has been suggested that changes in perceptions could be related to children’s poor understanding of the items, or children just have low commitment to completing the questionnaires, which can lead to contradictory interpretations and lower test-retest reliability [34].

### Limitations and strengths

When interpreting the results from this study, a few limitations need to be acknowledged. First, the readability scores for the Y-PASS questionnaires were appropriate for Grades 6 and 7, which is beyond the level of some of the participants in this study, who were in Grade 5. However, research assistants were available to assist participants and clarify any items that participants did not understand throughout all data collection session. While the sample size met some of the rules of thumb, it did not reach the frequently recommended 10:1 participant to variable ratio for each subscale, as suggested by Hair et al. [55]. Future studies reviewing the factorial structure of the Y-PASS questionnaires should aim to recruit a larger sample size, preferably greater than 300 participants [61]. The sampling method chosen for this study was to capture diversity of the school types. However, response rates did differ by school type, with fewer participants consenting from low SES schools compared to high SES schools. This may have resulted in some sampling bias effect in the analyses. The Cronbach alpha values for some of the factors (e.g. the lunchtime “Social barriers”, lunchtime “Physical environmental/policy barriers”, after-school “Perceived barriers” and after-school “Parental rules”) were below the acceptable level of internal consistency (i.e. Cronbach alpha of ≥0.6 [8,57]). This may have been due to the low number of items that loaded onto each of these factors. These factors were not removed from the questionnaires as the mean inter-item correlations did fit in the acceptable range of 0.10 to 0.50 [62]. Finally, it is recognised that factor analysis is usually conducted without a theoretical framework implied [56]. An initial exploratory factor analysis, with no theoretical framework inferred, produced uninterpretable factors, hence the decision to divide the items into theoretically-based subscales and then conduct an exploratory factor analysis within each subscale. While there is a level of theoretical bias, the resultant factors did fit conceptually with factors commonly identified in studies using a social ecological framework, confirming the appropriateness of imposing a theoretical framework onto the data. A similar approach has been adopted and reported in a number of questionnaire development studies [42-45].

## Conclusion

When surveying children, special attention should be paid to questionnaire construction and pretesting in order to optimise data quality [68]. Based on this preliminary reliability and factorial structure evidence, it can be concluded that these newly developed correlate questionnaires could be a useful tool for measuring potential correlates of context-specific physical activity in children aged 10–14 years. The multi-dimensional results from the exploratory factor analysis provide further justification for exploring physical activity correlates from a social ecological perspective and demonstrate the importance of developing items that are context specific. Testing the validity and reliability of any newly developed questionnaire is an ongoing process. Therefore, further development and refinement of the Y-PASS questionnaires is recommended, including a confirmatory factor analysis, exploring the inclusion of additional items and testing the questionnaires in samples outside of Australia where lunchtime and after-school settings may vary. Further, cross-sectional and experimental studies should be conducted to test the usefulness of the Y-PASS questionnaires in identifying the correlates of, and predictors of change, in lunchtime and after-school physical activity.

## Competing interests

The authors declare that they have no competing interests.

## Authors’ contributions

RMS contributed to the conceptualisation and design of the manuscript, collected and analysed data, and drafted the manuscript; KR, TO and JD contributed to the conceptualisation and design of the manuscript, interpretation of the data and provided substantive feedback on the manuscript. All authors read and approved the final manuscript.

## Pre-publication history

The pre-publication history for this paper can be accessed here:

http://www.biomedcentral.com/1471-2458/14/412/prepub

## Supplementary Material

Additional file 1 **List of potential correlate factors and correlate items for Draft Four of the lunchtime Y-PASS questionnaires [**[2]**,**[3]**,**[42]**,**[47]**,**[69]**-**[72]**].** Click here for file

Additional file 2 The lunchtime and after-school Y-PASS questionnaires. Click here for file
