# Supplementary material for: Development and psychometric properties of the Y-PASS questionnaire to assess correlates of lunchtime and after-school physical activity in children
Source: BMC Public Health. 2014 Apr 30;14:412. doi: 10.1186/1471-2458-14-412 (PMC4041362; doi:10.1186/1471-2458-14-412)
Supplement: Additional file 1 — List of potential correlate factors and correlate items for Draft Four of the lunchtime Y-PASS questionnaires [[2],[3],[42],[47],[69]-[72]]. [file 1471-2458-14-412-S1.docx]

Additional files

Additional file 1 - List of potential correlate factors and correlate items for Draft Four of the lunchtime Y-PASS questionnaires

| **Lunchtime Y-PASS** | **Items** | | **Source of items** | **Test-retest**  **(n = 24)** | **Cronbach alpha** | |
| --- | --- | --- | --- | --- | --- | --- |
| *Intrapersonal subscale* |  | |  |  |  | |
| Barrier self-efficacy | 1. I am confident that I can find other kids to be active with at lunchtime even if my friends don’t want to. 2. I am confident that I can be active at lunchtime even if the space in the playground/oval is limited. 3. I am confident that I can ask a teacher to get me equipment to play with at lunchtime. 4. I am confident that I can still be active at lunchtime even if my friends don’t want to. 5. I am confident that I can still be active in the school yard even if it is very hot or raining. 6. I am confident that I can ask my friends to be active with me during lunchtime. 7. I am confident that I can still be active at lunchtime even if there are bullies in the school yard. | | Items 1, 3, 4, 5, 6: modified from Saunders et al. [42].  Items 2-3: Purposely developed. | 0.84 | 0.80 | |
| Perceived self-efficacy | 1. I prefer to watch other kids rather than play active games at lunchtime.* 2. I am not good at being active at lunchtime.* 3. I prefer to sit rather than be active at lunchtime.* 4. There is nothing to do at lunchtime.* 5. It is fun to be active at lunchtime. 6. I have the skills I need to be active at lunchtime. | | Item 8: Modified from Ommundsen et al. [3].  Items 9-13: Purposely developed. | 0.73 | 0.78 | |
| Behavioural attitude/belief | 1. I am active at lunchtime so I can hang out with my friends. 2. I play certain games at lunchtime because I think I am good at them. 3. I am active at lunchtime because it makes me popular with the other children. 4. It is 'cool' to be active at lunchtime. 5. I play certain games at lunchtime because I want to get extra practice. | | Items 14, 15, 17, 18: Purposely developed.  Item 16: Modified from Saunders et al. [42]. | 0.73 | 0.78 | |
| Correlate items | 1. I can still be active at lunchtime even if I am wearing my school uniform. | | Purposely developed. | 0.67 | - | |
|  | 1. I like to walk around at lunchtime. | | Purposely developed. | 0.39 | - | |
|  | 1. I really like doing PE at school. | | Taken directly from Ommundsen et al. [3]. | 0.66 | - | |
|  | 1. I always have the energy to be active at lunchtime. | | Modified from Saunders et al. [42]. | 0.33 | - | |
|  | 1. I am just as coordinated as kids of my age and gender. | | Modified from Sallis et al. [47]. | 0.47 | - | |
|  | 1. Making up your own game rules makes playing games at lunchtime more fun. | | Purposely developed. | 0.47 | - | |
| *Sociocultural subscale* |  | |  |  |  | |
| Peer influence | 1. I teach other children how to play active games at lunchtime. 2. My friends teach me how to play active games at lunchtime. 3. I have friends who I am active with at lunchtime. | | All items purposely developed. | 0.70 | 0.64 | |
| Teacher influence | 1. Teachers help us with the active games we play at lunchtime. 2. Teachers play with us at lunchtime. | | All items purposely developed. | 0.63 | 0.64 | |
| Social barriers | 1. There is always a teacher who is on yard duty during lunchtime. 2. My friends would rather sit and talk at lunchtime.* 3. Bullying stops me from being active in the school yard at lunchtime.* | | All items purposely developed. | 0.57 | 0.32 | |
| *Physical environmental/policy subscale* | | |  |  |  | |
| Access to facilities/equipment | 1. There are lots of shaded areas where I can be active even if it is really hot. 2. There are indoor spaces where I can be active if it is raining. 3. There is enough equipment available for me to play with at lunchtime. | | All items purposely developed. | 0.71 | 0.61 | |
| Physical environmental/policy barriers | 1. It is hard to be active in our school uniform at lunchtime.* 2. The oval is too dry and hard to play on.* 3. Some school rules keep me from doing the activities I like at lunchtime.* | | All items purposely developed. | 0.66 | 0.55 | |
| Access to space | 1. There is enough space in the school yard for me to be active at lunchtime. 2. There is enough grass in the school yard to be active at lunchtime. 3. There are too many kids in the playground for me to be active at lunchtime.* | | All items purposely developed. | 0.60 | 0.50 | |
| Correlate items | 1. We have school rules about where we are allowed to be active at lunchtime.* | | Purposely developed. | 0.47 | - | |
|  | 1. Our school has areas that suit the games I want to play at lunchtime. | | Purposely developed. | 0.08 | - | |
|  | 1. There are facilities at school, such as playgrounds or ovals, where I can be active at lunchtime. | | Purposely developed. | 0.46 | - | |
|  | 1. Our school play area has painted lines on the ground (e.g. hopscotch and 4-square) to help me be active at lunchtime. | | Purposely developed. | 0.75 | - | |
| **After-school Y-PASS** | **Items** | **Source of items** | | **Test-retest**  **(n = 22)** | | **Cronbach alpha** |
| *Intrapersonal subscale* |  |  | |  | |  |
| Behavioural attitudes/beliefs (organised sports/activities) | 1. I don't do an organised sport or activity after school because other kids are better than me.* 2. I don't feel like doing an organised sport or activity after school.* 3. I enjoy being part of an organised sport or activity team. 4. I don't enjoy doing an organised sport or activity after school.* 5. It is not worth doing an organised sport or activity after school because I am not good at it.* 6. I am not active after school because I am scared that I will get injured.* 7. I prefer to watch other kids rather than do organised sports and activities after school.* | Items 1-6: Purposely developed.  Item 7: Modified from Ommundsen et al. [3]. | | 0.85 | | 0.77 |
| Behavioural attitudes/beliefs (non-organised activities) | 1. I prefer to be active after school instead of watching TV or playing electronic games. 2. Being active after school is the thing I like to do best. 3. I don't feel like playing actively at home or in the neighbourhood after school.* 4. Being active after school makes me feel good. 5. I am too tired to be active after school.* 6. It is fun being active after school. | Items 8, 10 11, 13: Purposely developed.  Item 9: Modified from Ommundsen et al. [3].  Item 12: Modified from Sallis et al. [47]. | | 0.93 | | 0.82 |
| Barriers self-efficacy | 1. I am confident that I can ask my parent or another adult to take me somewhere I can play actively after school. 2. I am confident that I can ask my parent or another adult to take me to an organised sport or activity after school. 3. I am confident that I can be active after school on most days even if I have to stay at home. 4. I am confident that I can be active after school on most days. 5. I am confident that I can ask friends to be active with me after school on most days. 6. I am confident that I can be active after school on most days no matter how busy I am. | Items 14-15: Modified from Saunders et al. [42].  Items 16-19: Taken directly from Motl et al. [69]. | | 0.73 | | 0.80 |
| Support seeking/social norm | 1. I play in the neighbourhood after school because I get to hang out with my friends. 2. I walk or ride to and from places after school because I get to hang out with my friends. 3. I play in the neighbourhood after school because I get to meet new people. | All items modified from Saunders et al. [42]. | | 0.58 | | 0.70 |
| Perceived competence | 1. I play active games after school because I think I am good at them. 2. I do an organised sport or activity after school because I think I am good at it. | All items purposely developed. | | 0.73 | | 0.82 |
| Perceived barriers | 1. I prefer to do homework rather than be active after school.* 2. I am scared of strangers in my neighbourhood after school.* 3. I am scared of dangerous animals in my yard, such as snakes, lizards, dogs or magpies.* | Items 25, 27: Purposely developed.  Item 26: Modified from Timperio et al. [70]. | | 0.62 | | 0.43 |
| Correlate items | 1. I wish I could do more organised sports or activities after school than I get a chance to. | Modified from Ommundsen et al. [3]. | | 0.72 | | - |
|  | 1. Being active after school is boring.* | Purposely developed. | | 0.63 | | - |
|  | 1. I do an organised sport or activity after school because I want to improve my skills. | Purposely developed. | | 0.68 | | - |
|  | 1. I do an organised sport or activity after school because I want to meet new people. | Purposely developed. | | 0.55 | | - |
|  | 1. I ride, walk, skate or scooter to and from places after school because it gets you fit. | Purposely developed. | | 0.37 | | - |
|  | 1. Playing outside after school keeps you healthy. | Modified from Robbins et al. [71]. | | 0.69 | | - |
|  | 1. I do an organised sport or activity after school because I have nothing else to do. | Purposely developed. | | 0.13 | | - |
|  | 1. I don’t participate in some activities after school because it is only a boys’/girls’ activity.* | Purposely developed. | | 0.26 | | - |
|  | 1. I play actively at home or in the neighbourhood after school because it gives me something to do. | Purposely developed. | | 0.57 | | - |
|  | 1. I do an organised sport or activity after school because it gets you fit. | Purposely developed. | | 0.45 | | - |
|  | 1. I am active after school because I don’t want to put on weight. | Purposely developed. | | 0.65 | | - |
|  | 1. I am confident that I can be active after school on most days even if I could watch TV or play video games instead. | Taken directly from Motl et al. [69] | | 0.48 | | - |
|  | 1. I am confident that I can be active after school on most days even if it is hot or cold outside. | Taken directly from Motl et al. [69] | | 0.66 | |  |
|  | 1. I am confident that I can be active after school on most days even if I am being bullied. | Purposely developed. | | 0.72 | |  |
|  | 1. I am just as coordinated as kids of my age and gender. | Modified from Sallis et al. [47]. | | 0.80 | |  |
|  | 1. I am shy about how my body looks.* | Purposely developed. | | 0.69 | |  |
|  | 1. I really like doing PE at school. | Taken directly from Ommundsen et al. [3]. | | 0.57 | |  |
| *Sociocultural subscale* |  |  | |  | |  |
| Social support | 1. My parents help me practise sport after school. 2. My parents play actively with me after school. 3. My family tell me I am doing well at my after-school organised sport or activity. 4. My family always watch me do an organised sport or activity after school. 5. My parents encourage me to play outside after school. 6. My parents encourage me to do an organised sport or activity after school. 7. I do an organised sport or activity with friends after school. | Items 45, 46, 48, 50: Modified from Ommundsen et al. [3].  Item 47: Modified from Hohepa et al. [2].  Items 49, 51: Purposely developed. | | 0.91 | | 0.78 |
| Parental barriers | 1. I don’t do an organised sport or activity after school because my parents work late.* 2. I am not allowed to do an organised sport or activity after school because my parents are scared that I might get hurt.* 3. My parents won’t let me do an organised sport or activity because I am already doing too many other activities.* 4. My parents are not home after school to supervise my play.* | Items 52-54: Purposely developed.  Item 54: Modified from Rushovich et al. [72]. | | 0.55 | | 0.63 |
| License to be active | 1. My parents won’t let me ride, walk, skate or scooter to and from places after school.* 2. I walk, ride, skate or scooter to and from places with friends after school. 3. My parents think it is safe for me to be active in the neighbourhood after school. 4. I play with friends in the neighbourhood after school. | Item 56: Purposely developed.  Item 57: Modified from Saunders et al. [42].  Item 58: Modified from Timperio et al. [70].  Item 59: Modified from Ommundsen et al. [3]. | | 0.85 | | 0.75 |
| Parental rules | 1. If I am going out after school, I always have to be back by a certain time.* 2. I always have to tell my parents where I am when I go out after school.* | All items taken directly from Ommundsen et al. [3]. | | 0.77 | | 0.56 |
| Correlate items | 1. Bullying stops me from being active after school.* | Purposely developed. | | 0.34 | | - |
|  | 1. I am not active after school because I have no one to play with.* | Purposely developed. | | 0.75 | | - |
|  | 1. I have brothers/sisters who play actively with me after school. | Purposely developed. | | 0.41 | | - |
|  | 1. My friends encourage me to be active after school. | Modified from Saunders et al. [42]. | | 0.33 | | - |
|  | 1. My friends tell me I am doing well at sport. | Modified from Hohepa et al. [2]. | | 0.60 | | - |
|  | 1. My parents are too busy to play with me after school.* | Purposely developed. | | 0.58 | | - |
|  | 1. My parents make me help around the house, which stops me from being active after school.* | Purposely developed. | | 0.31 | | - |
|  | 1. We have a rule at home that we have to do an organised sport or activity after school. | Purposely developed. | | 0.40 | | - |
| *Physical environmental/policy subscale* | | | | | | |
| Weather | 1. When it is too hot, it stops me from playing actively after school.* 2. When it is raining, it stops me from playing actively after school.* 3. When it is raining, it stops me from walking, riding, skating or riding a scooter to and from places after school.* 4. When it is too hot, it stops me from walking, riding, skating or riding a scooter to and from places after school.* 5. When it is too hot, it stops me from doing an organised sport or activity after school.* 6. When it is raining, it stops me from doing an organised sport or activity after school.* | All items purposely developed. | | 0.69 | | 0.75 |
| Access to facilities/equipment | 1. It is easy to get to an organised sport or activity after school. 2. There are playgrounds or parks near my house where I can be active after school. 3. I don’t have to travel far to play with my friends after school. 4. There are sport or recreation centres that I can go to after school. 5. I have the right equipment to do my chosen organised sport or activity after school. 6. I live too far away to walk, ride, skate or scooter to and from places after school.* 7. I have the right equipment (e.g. a bike lock, helmet or bike) to ride a bike after school. | Item 76, 79-82: Purposely developed.  Item 77: Modified from Saunders et al. [42].  Item 78: Modified from Ommundsen et al. [3]. | | 0.80 | | 0.69 |
| Safety | 1. The roads are safe in my neighbourhood after school. 2. There is heavy traffic in the streets where I live.* 3. There are dangerous objects in my yard, such as rusty scrap metal.* 4. It is safe to play actively in my yard after school. | Items 83-84: Modified from Timperio et al. [70].  Item 85: Purposely developed.  Item 86: Modified from Ommundsen et al. [3]. | | 0.75 | | 0.63 |
| Access to space | 1. My yard is too small for me to be active after school.* 2. I play actively in my yard after school because I have a lot of lawn. 3. There is somewhere at home where I can play actively after school. | All items purposely developed. | | 0.75 | | 0.60 |
| Time commitments | 1. Homework stops me from doing an organised sport or activity after school.* 2. Homework stops me from playing actively at home or in the neighbourhood after school.* | All items modified from Sallis et al. [47]. | | 0.66 | | 0.61 |
| Financial barriers | 1. We do not have enough cars to drive to and from places where I can be active after school.* 2. Petrol costs too much to drive to and from places where I can be active after school.* 3. It costs too much money to do an organised sport or activity after school.* | Item 92, 94: Purposely developed.  Item 93: Modified from Ommundsen et al. [3]. | | 0.60 | | 0.64 |
| School bag | 1. My school bag(s) is too heavy for me to walk, ride, skate or scooter home after school.* 2. I don’t walk, ride, skate or scooter home from school when I have too many bags to carry.* | All items purposely developed. | | 0.53 | | 0.54 |
| Correlate items | 1. Dog poo on the lawn at home keeps me from being active on the grass after school.* | Purposely developed. | | 0.38 | | - |
|  | 1. I have enough time to do an organised sport or activity after school. | Modified from Sallis et al. [47]. | | 0.80 | | - |
|  | 1. It is safe to play actively near where I live after school. | Modified from Ommundsen et al. [3]. | | 0.70 | | - |
|  | 1. There are not enough traffic lights and crossings in my neighbourhood after school.* | Modified from Timperio et al. [70] | | 0.47 | | - |
|  | 1. There is enough equipment to play actively at home after school. | Modified from Saunders et al. [42]. | | 0.27 | | - |
| *Notes*: * = items that are reverse coded. | | | |  | |  |
