# Supplementary material for: Development and psychometric properties of the Y-PASS questionnaire to assess correlates of lunchtime and after-school physical activity in children
Source: BMC Public Health. 2014 Apr 30;14:412. doi: 10.1186/1471-2458-14-412 (PMC4041362; doi:10.1186/1471-2458-14-412)
Supplement: Additional file 2 — The lunchtime and after-school Y-PASS questionnaires. [file 1471-2458-14-412-S2.docx]

Additional files

Additional file 2 – The lunchtime and after-school Y-PASS questionnaires

**Y-PASS: The Youth Physical Activity Survey for Specific Settings**

Y-PASS: The Youth Physical Activity Survey for Specific Settings

**The Lunchtime Questionnaire**

**
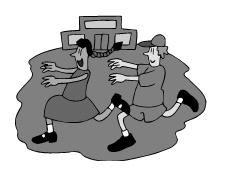
**

What is your ID number? (this is the number on the piece of paper titled "Y-PASS Questionnaire")*

____________________________________________

INTRODUCTION

Thank you for agreeing to fill out this important questionnaire.


The information you share will help us to understand what things help children be active or make it difficult for children to be active during different times of the day.

**• Please answer every question carefully.**

**• Do not spend too much time on one question.**

**• If something is not clear, please ask for help.**

**• This is NOT a test.**

**• There are no right or wrong answers.**

**• We are interested in what you think, so please be as honest as you can in your responses.


Thank you for your help with this project.**

LET'S BEGIN THE QUESTIONNAIRE WITH SOME INFORMATION ABOUT YOU

**Please fill out the following information**

1) What is your first name?*

____________________________________________

2) What is your last name?*

____________________________________________

3) What is the name of your school?*

____________________________________________

4) What year are you in?*

( ) Year 5

( ) Year 6

( ) Year 7

5) Are you a boy or girl? Please tick:*

( ) Boy

( ) Girl

6) What is your Date of Birth? (dd/mm/yyyy, e.g. 02/10/1990)*

____________________________________________

7) In what country were you born?*

( ) Australia

( ) Another Country (please specify): _________________

8) Are you an Aboriginal or Torres Strait Islander? Please tick:*

( ) Yes

( ) No

9) What suburb/town do you live in?*

____________________________________________

10) What is the postcode where you live?

____________________________________________

DEFINITION OF TERMS

**Before you move on, it is important to understand some of the words used in this questionnaire.**

**BEING ACTIVE involves any movement which can make you breathe quicker and heavier than usual. You may start to sweat and feel out of breath.**

**There are different ways of BEING ACTIVE at lunchtime, including:**

Playing four-square

Playing on the playground

Playing on the oval

Skipping

Playing chasey

**
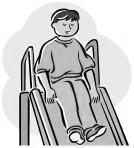

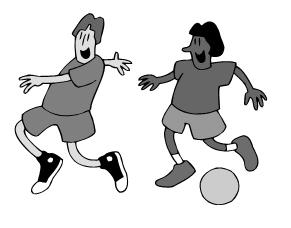

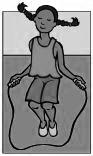
**

**
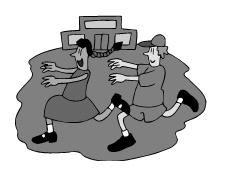
**LET’S START THINKING ABOUT LUNCHTIME AT SCHOOL

**In a USUAL WEEK, what do you do at lunchtime?**

**You may want to think about the following things:**

What games do you play at lunchtime?

Who do you hang out with at lunchtime?

Where do you normally hang out at lunchtime?

Tick the **MAIN ACTIVITIES** that you usually do at lunchtime and how often you do these activities at lunchtime. You can pick more than one activity.

**Example:**


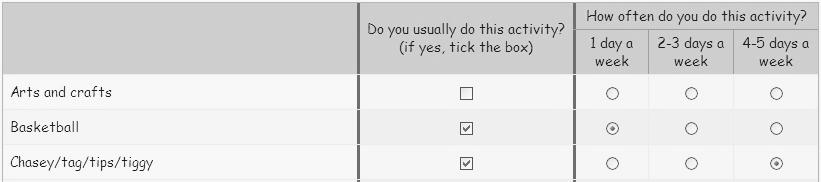


|  | **Do you usually do this activity? (if yes, tick the box)** | **How often do you do this activity?** | | |
| --- | --- | --- | --- | --- |
|  | **Yes** | **1 day a week** | **2-3 days a week** | **4-5 days a week** |
| Arts and crafts | [ ] | ( ) | ( ) | ( ) |
| Basketball | [ ] | ( ) | ( ) | ( ) |
| Chasey/tag/tips/tiggy | [ ] | ( ) | ( ) | ( ) |
| Computer work (e.g. typing/internet) | [ ] | ( ) | ( ) | ( ) |
| Cricket | [ ] | ( ) | ( ) | ( ) |
| Dodge ball/poison ball/brandy/speed ball | [ ] | ( ) | ( ) | ( ) |
| Football (Australian/Gaelic/American) | [ ] | ( ) | ( ) | ( ) |
| Hand tennis (four-square) | [ ] | ( ) | ( ) | ( ) |
| Kickball | [ ] | ( ) | ( ) | ( ) |
| Mucking around outdoors in the school yard | [ ] | ( ) | ( ) | ( ) |
| Netball | [ ] | ( ) | ( ) | ( ) |
| Playing catch | [ ] | ( ) | ( ) | ( ) |
| Playing on playground equipment (e.g. monkey bars) | [ ] | ( ) | ( ) | ( ) |
| Rugby league | [ ] | ( ) | ( ) | ( ) |
| Running/jogging | [ ] | ( ) | ( ) | ( ) |
| Skipping/jump rope | [ ] | ( ) | ( ) | ( ) |
| Sitting (e.g. eating, talking to friends, lunchtime meetings, writing, reading) | [ ] | ( ) | ( ) | ( ) |
| Soccer (field/indoor) | [ ] | ( ) | ( ) | ( ) |
| Touch football | [ ] | ( ) | ( ) | ( ) |
| Walking around the school yard | [ ] | ( ) | ( ) | ( ) |

Do you **USUALLY** do activities at lunchtime that are **NOT** on the list?

If **NO**, please click "next" to continue with the questionnaire.

If **YES**, please complete the table below.

|  | **Name the activity** | **How often do you do these activities at lunchtime?** | | |
| --- | --- | --- | --- | --- |
|  |  | **1 day a week** | **2-3 days a week** | **4-5 days a week** |
| 1. Other |  | ( ) | ( ) | ( ) |
| 2. Other |  | ( ) | ( ) | ( ) |
| 3. Other |  | ( ) | ( ) | ( ) |
| 4. Other |  | ( ) | ( ) | ( ) |
| 5. Other |  | ( ) | ( ) | ( ) |

Think about where you play at school at lunchtime

**Please click on the circle which most closely describes how much you agree or disagree with each statement.**


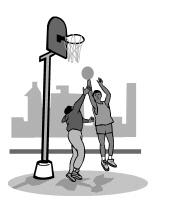
11) There is enough space in the school yard for me to be active at lunchtime.*

( ) Disagree a lot

( ) Disagree a little

( ) Neither disagree nor agree

( ) Agree a little

( ) Agree a lot

12) We have school rules about where we are allowed to be active at lunchtime.*

( ) Disagree a lot

( ) Disagree a little

( ) Neither disagree nor agree

( ) Agree a little

( ) Agree a lot

13) Our school has areas that suit the games I want to play at lunchtime.*

( ) Disagree a lot

( ) Disagree a little

( ) Neither disagree nor agree

( ) Agree a little

( ) Agree a lot

14) There are too many kids in the playground for me to be active at lunchtime.*

( ) Disagree a lot

( ) Disagree a little

( ) Neither disagree nor agree

( ) Agree a little

( ) Agree a lot

15) There are lots of shaded areas where I can be active even if it is really hot.*

( ) Disagree a lot

( ) Disagree a little

( ) Neither disagree nor agree

( ) Agree a little

( ) Agree a lot

16) There are indoor spaces where I can be active if it is raining.*

( ) Disagree a lot

( ) Disagree a little

( ) Neither disagree nor agree

( ) Agree a little

( ) Agree a lot

17) The oval is too dry and hard to play on.*

( ) Disagree a lot

( ) Disagree a little

( ) Neither disagree nor agree

( ) Agree a little

( ) Agree a lot

18) There is enough grass in the school yard to be active at lunchtime.*

( ) Disagree a lot

( ) Disagree a little

( ) Neither disagree nor agree

( ) Agree a little

( ) Agree a lot

19) Our school play area has painted lines on the ground (e.g. hopscotch and 4-square) to help me be active at lunchtime.*

( ) Disagree a lot


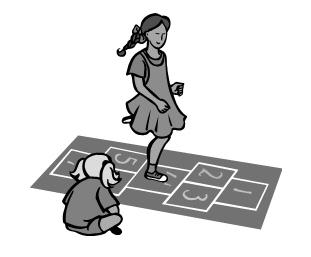
( ) Disagree a little

( ) Neither disagree nor agree

( ) Agree a little

( ) Agree a lot

20) There are facilities at school, such as playgrounds or ovals, where I can be active at lunchtime.*

( ) Disagree a lot

( ) Disagree a little

( ) Neither disagree nor agree

( ) Agree a little

( ) Agree a lot

Still thinking about when you are active in the school yard at lunchtime, please click on the circle which most closely describes how much you agree or disagree with each statement.

21) It is hard to be active in our school uniform at lunchtime.*

( ) Disagree a lot

( ) Disagree a little

( ) Neither disagree nor agree

( ) Agree a little

( ) Agree a lot

22) There is enough equipment available for me to play with at lunchtime.*

( ) Disagree a lot


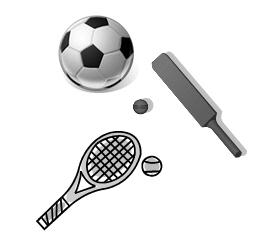
( ) Disagree a little

( ) Neither disagree nor agree

( ) Agree a little

( ) Agree a lot

23) Some school rules keep me from doing the activities I like at lunchtime.*

( ) Disagree a lot

( ) Disagree a little

( ) Neither disagree nor agree

( ) Agree a little

( ) Agree a lot

24) I have other commitments at lunchtime that keep me from being active, such as meetings and music lessons.*

( ) Disagree a lot

( ) Disagree a little

( ) Neither disagree nor agree

( ) Agree a little

( ) Agree a lot

25) I can still be active at lunchtime even if I am wearing my school uniform.*

( ) Disagree a lot

( ) Disagree a little

( ) Neither disagree nor agree

( ) Agree a little

( ) Agree a lot

The next section is about your enjoyment of being active

**Please click on the circle which most closely describes how much you agree or disagree with each statement.**


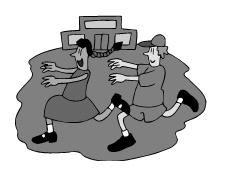
26) It is fun to be active at lunchtime.*

( ) Disagree a lot

( ) Disagree a little

( ) Neither disagree nor agree

( ) Agree a little

( ) Agree a lot

27) I prefer to watch other kids rather than play active games at lunchtime.*

( ) Disagree a lot

( ) Disagree a little

( ) Neither disagree nor agree


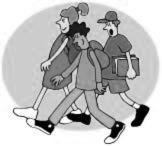
( ) Agree a little

( ) Agree a lot

28) I like to walk around at lunchtime.*

( ) Disagree a lot

( ) Disagree a little

( ) Neither disagree nor agree

( ) Agree a little

( ) Agree a lot

29) I prefer to sit rather than be active at lunchtime.*

( ) Disagree a lot

( ) Disagree a little

( ) Neither disagree nor agree

( ) Agree a little

( ) Agree a lot

30) I really like doing PE at school.*

( ) Disagree a lot

( ) Disagree a little

( ) Neither disagree nor agree

( ) Agree a little

( ) Agree a lot

Still thinking about lunchtime at school, please click on the circle which most closely describes how much you agree or disagree with each statement.

31) Making up your own game rules makes playing games at lunchtime more fun.*

( ) Disagree a lot

( ) Disagree a little

( ) Neither disagree nor agree

( ) Agree a little

( ) Agree a lot

32) I am active at lunchtime because it makes me popular with the other children.*

( ) Disagree a lot

( ) Disagree a little


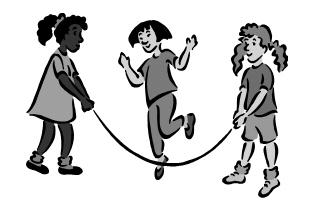
( ) Neither disagree nor agree

( ) Agree a little

( ) Agree a lot

33) I always have the energy to be active at lunchtime.*

( ) Disagree a lot

( ) Disagree a little

( ) Neither disagree nor agree

( ) Agree a little

( ) Agree a lot

34) I play certain games at lunchtime because I want to get extra practice.*

( ) Disagree a lot

( ) Disagree a little

( ) Neither disagree nor agree

( ) Agree a little

( ) Agree a lot

35) I am active at lunchtime so I can hang out with my friends.*

( ) Disagree a lot

( ) Disagree a little

( ) Neither disagree nor agree

( ) Agree a little

( ) Agree a lot

36) There is nothing to do at lunchtime.*

( ) Disagree a lot

( ) Disagree a little

( ) Neither disagree nor agree

( ) Agree a little

( ) Agree a lot

37) It is 'cool' to be active at lunchtime.*

( ) Disagree a lot

( ) Disagree a little

( ) Neither disagree nor agree

( ) Agree a little

( ) Agree a lot

38) I don't play in certain areas of the school yard because I am scared of the other kids.*

( ) Disagree a lot

( ) Disagree a little

( ) Neither disagree nor agree

( ) Agree a little

( ) Agree a lot

The following statements are about your confidence to be active at lunchtime

Please click on the circle which most closely describes **how much you agree or disagree with each statement**.


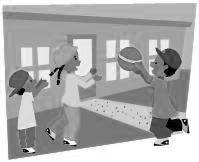
39) I am confident that I can ask my friends to be active with me during lunchtime.*

( ) Disagree a lot

( ) Disagree a little

( ) Neither disagree nor agree

( ) Agree a little

( ) Agree a lot

40) I am confident that I can find other kids to be active with at lunchtime even if my friends don't want to.*

( ) Disagree a lot

( ) Disagree a little

( ) Neither disagree nor agree

( ) Agree a little

( ) Agree a lot

41) I am confident that I can ask a teacher to get me equipment to play with at lunchtime.*

( ) Disagree a lot

( ) Disagree a little

( ) Neither disagree nor agree

( ) Agree a little

( ) Agree a lot

42) I am confident that I can be active at lunchtime even if the space in the playground/oval is limited.*

( ) Disagree a lot

( ) Disagree a little

( ) Neither disagree nor agree

( ) Agree a little

( ) Agree a lot

43) I am confident that I can still be active in the school yard even if it is very hot or raining.*

( ) Disagree a lot

( ) Disagree a little

( ) Neither disagree nor agree

( ) Agree a little

( ) Agree a lot

44) I am confident that I can still be active at lunchtime even if there are bullies in the school yard.*

( ) Disagree a lot

( ) Disagree a little


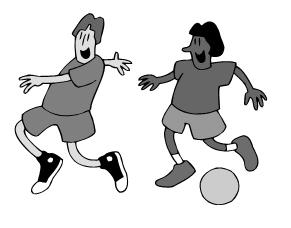
( ) Neither disagree nor agree

( ) Agree a little

( ) Agree a lot

45) I am confident that I can still be active at lunchtime even if my friends don't want to.*

( ) Disagree a lot

( ) Disagree a little

( ) Neither disagree nor agree

( ) Agree a little

( ) Agree a lot

Now think about your friends and the people around you at lunchtime

**Please click on the circle which most closely describes how much you agree or disagree with each statement.**

46) Bullying stops me from being active in the school yard at lunchtime.*

( ) Disagree a lot

( ) Disagree a little

( ) Neither disagree nor agree

( ) Agree a little

( ) Agree a lot

47) My friends would rather sit and talk at lunchtime.*

( ) Disagree a lot

( ) Disagree a little

( ) Neither disagree nor agree

( ) Agree a little

( ) Agree a lot

48) I have friends who I am active with at lunchtime.*

( ) Disagree a lot

( ) Disagree a little

( ) Neither disagree nor agree

( ) Agree a little

( ) Agree a lot

49) My friends teach me how to play active games at lunchtime.*

( ) Disagree a lot

( ) Disagree a little

( ) Neither disagree nor agree

( ) Agree a little

( ) Agree a lot

50) I do what my friends do at lunchtime so I don't feel left out.*

( ) Disagree a lot

( ) Disagree a little

( ) Neither disagree nor agree

( ) Agree a little

( ) Agree a lot

51) I teach other children how to play active games at lunchtime.*

( ) Disagree a lot

( ) Disagree a little

( ) Neither disagree nor agree

( ) Agree a little

( ) Agree a lot

52) I only play active games if other children/friends organise it.*

( ) Disagree a lot

( ) Disagree a little

( ) Neither disagree nor agree

( ) Agree a little

( ) Agree a lot

Thinking about the teachers at your school, please click on the circle which most closely describes how much you agree or disagree with each statement.

53) Teachers play with us at lunchtime.*

( ) Disagree a lot

( ) Disagree a little

( ) Neither disagree nor agree

( ) Agree a little

( ) Agree a lot

54) There is always a teacher who is on yard duty during lunchtime.*

( ) Disagree a lot

( ) Disagree a little

( ) Neither disagree nor agree

( ) Agree a little

( ) Agree a lot

55) Teachers help us with the active games we play at lunchtime.*

( ) Disagree a lot

( ) Disagree a little

( ) Neither disagree nor agree

( ) Agree a little

( ) Agree a lot

56) Teachers encourage us to be active at lunchtime.*

( ) Disagree a lot

( ) Disagree a little

( ) Neither disagree nor agree

( ) Agree a little

( ) Agree a lot

The next statements are about how good you think you are at playing active games at lunchtime

Please click on the circle which most closely describes **how much you agree or disagree with each statement.**

57) I play certain games at lunchtime because I think I am good at them.*

( ) Disagree a lot

( ) Disagree a little

( ) Neither disagree nor agree

( ) Agree a little

( ) Agree a lot

58) I have the skills I need to be active at lunchtime.*

( ) Disagree a lot

( ) Disagree a little

( ) Neither disagree nor agree

( ) Agree a little

( ) Agree a lot


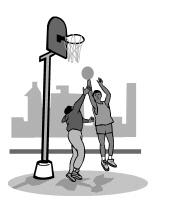


59) I am **NOT** good at being active at lunchtime.*

( ) Disagree a lot

( ) Disagree a little

( ) Neither disagree nor agree

( ) Agree a little

( ) Agree a lot

60) I am just as coordinated as kids of my age and gender.*

( ) Disagree a lot

( ) Disagree a little

( ) Neither disagree nor agree

( ) Agree a little

( ) Agree a lot

Thank You!

Thank you for completing this survey.

**Y-PASS: The Youth Physical Activity Survey for Specific Settings**

Y-PASS: The Youth Physical Activity Survey for Specific Settings

**The After School Questionnaire**

**
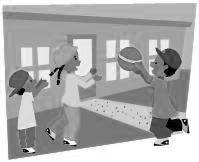
**

What is your ID number? (this is the number on the piece of paper titled "Y-PASS Questionnaire")*

____________________________________________

INTRODUCTION

Thank you for agreeing to fill out this important questionnaire.


The information you share will help us to understand what things help children be active or make it difficult for children to be active during different times of the day.

**• Please answer every question carefully.**

**• Do not spend too much time on one question.**

**• If something is not clear, please ask for help.**

**• This is NOT a test.**

**• There are no right or wrong answers.**

**• We are interested in what you think, so please be as honest as you can in your responses.


Thank you for your help with this project.**

LET’S BEGIN THE QUESTIONNAIRE WITH SOME INFORMATION ABOUT YOU

Please fill out the following information

1) What is your first name?*

____________________________________________

2) What is your last name?*

____________________________________________

3) What is the name of your school?*

____________________________________________

4) What year are you in?*

( ) Year 5

( ) Year 6

( ) Year 7

5) Are you a boy or girl? Please tick:*

( ) Boy

( ) Girl

6) What is your Date of Birth? (dd/mm/yyyy, e.g. 05/09/1991)*

____________________________________________

7) In what country were you born?*

( ) Australia

( ) Another Country (please specify): _________________

8) Are you an Aboriginal or Torres Strait Islander? Please tick:*

( ) Yes

( ) No

9) What suburb/town do you live in?*

____________________________________________

10) What is the postcode where you live?

____________________________________________

DEFINITION OF TERMS

**Before you move on, it is important to understand some of the words used in this questionnaire.**

**BEING ACTIVE involves any movement which can make you breathe quicker and heavier than usual. You may start to sweat and feel out of breath.

There are different ways of BEING ACTIVE, including: Active transportation; Non-organised play, games and activities; and Organised sports and activities. You can be active at school, home or in your neighbourhood.**

**ACTIVE TRANSPORTATION is how you get to and from places actively (i.e. not using motorised transport like a car or bus). Some examples of active transportation include:**

Walking or riding your bike, skateboard or scooter home from school

Walking or riding your bike, skateboard or scooter to and from places in your neighbourhood

Walking or riding your bike, skateboard or scooter to sport or activity practices

**
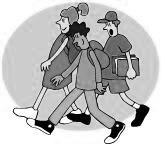

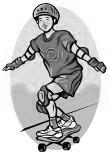

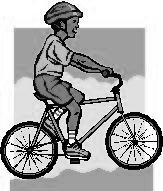
**

**NON-ORGANISED PLAY, GAMES AND ACTIVITIES are NOT led by an adult. Some examples of being active in non-organised play, games and activities include:**

Playing four-square with your friends

Playing in the playground

Taking the dog for a walk

Mucking around with your friends or siblings

Riding your bike, skateboard or scooter (not for transport)

Jumping on the trampoline

**
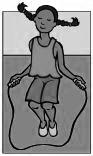

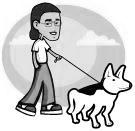

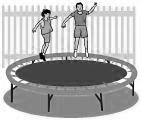

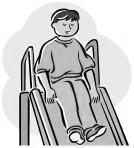

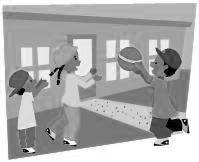
**

**ORGANISED SPORTS AND ACTIVITIES are directed by a leader, such as a coach or a teacher. Some examples of organised sports and activities include:**

Scouts

Girl Guides

Dancing

Any sport or sport practices, such as soccer, football, netball, swimming and basketball

**
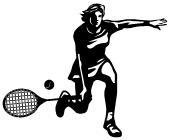

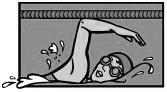

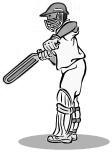

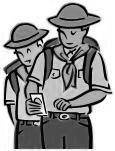

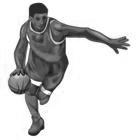

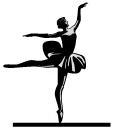
**

LET’S START THINKING ABOUT AFTER SCHOOL

**This is the period from when the home time school bell goes to just before dinner.**


In a **USUAL WEEK**, what do you do after school?

**You may want to think about the following things:**

What organised sports or activities do you do after school?

What non-organised play, games or activities do you do after school?


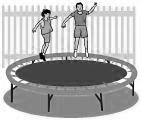


Who do you hang out with after school?

Where do you normally go after school?

Tick the **MAIN ACTIVITIES** that you usually do after school and how often you do these activities after school. You can pick more than one activity.

**Example:**


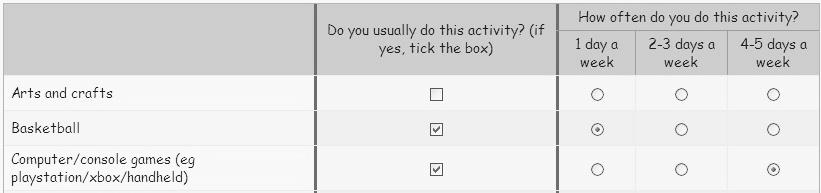


|  | **Do you usually do this activity? (if yes, tick the box)** | **How often do you do this activity?** | | |
| --- | --- | --- | --- | --- |
|  | **Yes** | **1 day a week** | **2-3 days a week** | **4-5 days a week** |
| Arts and crafts | [ ] | ( ) | ( ) | ( ) |
| Basketball | [ ] | ( ) | ( ) | ( ) |
| Computer/console games (e.g. playstation/xbox/handheld) | [ ] | ( ) | ( ) | ( ) |
| Computer work (e.g. typing/internet) | [ ] | ( ) | ( ) | ( ) |
| Dancing (general) | [ ] | ( ) | ( ) | ( ) |
| Dressing and undressing | [ ] | ( ) | ( ) | ( ) |
| Feeding or caring for pets/farm animals | [ ] | ( ) | ( ) | ( ) |
| Football (Australian/Gaelic/American) | [ ] | ( ) | ( ) | ( ) |
| Kickball | [ ] | ( ) | ( ) | ( ) |
| Mucking around indoors walk/run | [ ] | ( ) | ( ) | ( ) |
| Mucking around outdoors | [ ] | ( ) | ( ) | ( ) |
| Netball | [ ] | ( ) | ( ) | ( ) |
| Packing/unpacking bag | [ ] | ( ) | ( ) | ( ) |
| Playing with animals (e.g. throwing a ball to your dog) | [ ] | ( ) | ( ) | ( ) |
| Playing cards/puzzles/board games | [ ] | ( ) | ( ) | ( ) |
| Playing the piano or organ | [ ] | ( ) | ( ) | ( ) |
| Riding a bicycle/bike | [ ] | ( ) | ( ) | ( ) |
| Riding in a bus/train/tram | [ ] | ( ) | ( ) | ( ) |
| Riding in a car/truck | [ ] | ( ) | ( ) | ( ) |
| Running/jogging | [ ] | ( ) | ( ) | ( ) |
| Shopping | [ ] | ( ) | ( ) | ( ) |
| Sitting (e.g. talking, eating, reading) | [ ] | ( ) | ( ) | ( ) |
| Soccer (field/indoor) | [ ] | ( ) | ( ) | ( ) |
| Studying/homework or writing | [ ] | ( ) | ( ) | ( ) |
| Swimming laps | [ ] | ( ) | ( ) | ( ) |
| Tidying/cleaning room | [ ] | ( ) | ( ) | ( ) |
| Trampoline | [ ] | ( ) | ( ) | ( ) |
| Walking | [ ] | ( ) | ( ) | ( ) |
| Walking the dog | [ ] | ( ) | ( ) | ( ) |
| Watching TV | [ ] | ( ) | ( ) | ( ) |

Do you **USUALLY** do activities after school that are **NOT** on the list?

If **NO**, please click "next" to continue with the questionnaire.

If **YES**, please complete the table below.

|  | **Name the activity** | **How often do you do these activities after school?** | | |
| --- | --- | --- | --- | --- |
|  |  | **1 day a week** | **2-3 days a week** | **4-5 days a week** |
| 1. Other |  | ( ) | ( ) | ( ) |
| 2. Other |  | ( ) | ( ) | ( ) |
| 3. Other |  | ( ) | ( ) | ( ) |
| 4. Other |  | ( ) | ( ) | ( ) |
| 5. Other |  | ( ) | ( ) | ( ) |

Think about the places where you are active after school

**Please click on the circle which most closely describes how much you agree or disagree with each statement.**

11) There is somewhere at home where I can play actively after school.*


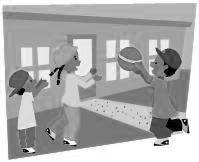
( ) Disagree a lot

( ) Disagree a little

( ) Neither disagree nor agree

( ) Agree a little

( ) Agree a lot

12) My yard is too small for me to be active after school.*

( ) Disagree a lot

( ) Disagree a little

( ) Neither disagree nor agree

( ) Agree a little

( ) Agree a lot

13) Dog poo on the lawn at home keeps me from being active on the grass after school.*

( ) Disagree a lot


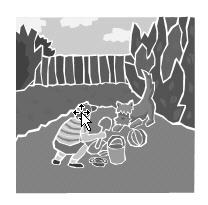
( ) Disagree a little

( ) Neither disagree nor agree

( ) Agree a little

( ) Agree a lot

14) I play actively in my yard after school because I have a lot of lawn.*

( ) Disagree a lot

( ) Disagree a little

( ) Neither disagree nor agree

( ) Agree a little

( ) Agree a lot

The next section is about the distance to and from places after school

Please click on the circle which most closely describes **how much you agree or disagree with each statement.**

15) I don't have to travel far to play with my friends after school.*

( ) Disagree a lot

( ) Disagree a little

( ) Neither disagree nor agree

( ) Agree a little

( ) Agree a lot

16) I live too far away to walk, ride, skate or scooter to and from places after school.*

( ) Disagree a lot

( ) Disagree a little

( ) Neither disagree nor agree

( ) Agree a little

( ) Agree a lot

17) I do an organised sport or activity because it is close to where I live.*

( ) Disagree a lot

( ) Disagree a little

( ) Neither disagree nor agree

( ) Agree a little

( ) Agree a lot

The following statements are about cost

Please click on the circle which most closely describes **how much you agree or disagree with each statement.**

18) Petrol costs too much to drive to and from places where I can be active after school.*

( ) Disagree a lot

( ) Disagree a little

( ) Neither disagree nor agree

( ) Agree a little

( ) Agree a lot

19) We do not have enough cars to drive to and from places where I can be active after school.*

( ) Disagree a lot

( ) Disagree a little

( ) Neither disagree nor agree

( ) Agree a little

( ) Agree a lot

20) It costs too much money to do an organised sport or activity after school.*

( ) Disagree a lot

( ) Disagree a little

( ) Neither disagree nor agree

( ) Agree a little

( ) Agree a lot

Think about the activities you do after school

Please click on the circle which most closely describes **how much you agree or disagree with each statement.**

21) There are playgrounds or parks near my house where I can be active after school.*

( ) Disagree a lot

( ) Disagree a little

( ) Neither disagree nor agree


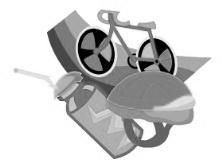
( ) Agree a little

( ) Agree a lot

22) I have the right equipment (e.g. a bike lock, helmet or bike) to ride a bike after school.*

( ) Disagree a lot

( ) Disagree a little

( ) Neither disagree nor agree

( ) Agree a little

( ) Agree a lot

23) I have the right equipment to do my chosen organised sport or activity after school.*

( ) Disagree a lot

( ) Disagree a little

( ) Neither disagree nor agree

( ) Agree a little

( ) Agree a lot

24) It is easy to get to an organised sport or activity after school.*

( ) Disagree a lot

( ) Disagree a little

( ) Neither disagree nor agree


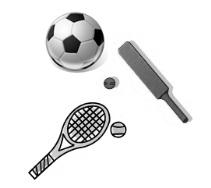
( ) Agree a little

( ) Agree a lot

25) There are sport or recreation centres that I can go to after school.*

( ) Disagree a lot

( ) Disagree a little

( ) Neither disagree nor agree

( ) Agree a little

( ) Agree a lot

26) There is enough equipment to play actively at home after school.*

( ) Disagree a lot

( ) Disagree a little

( ) Neither disagree nor agree

( ) Agree a little

( ) Agree a lot

Still thinking about after school, please click on the circle which most closely describes how much you agree or disagree with each statement.

27) It is safe to play actively near where I live after school.*

( ) Disagree a lot

( ) Disagree a little

( ) Neither disagree nor agree

( ) Agree a little

( ) Agree a lot

28) It is safe to play actively in my yard after school.*

( ) Disagree a lot

( ) Disagree a little

( ) Neither disagree nor agree

( ) Agree a little

( ) Agree a lot

Think about your neighbourhood after school

**Please click on the circle which most closely describes how much you agree or disagree with each statement.**

29) I am scared of strangers in my neighbourhood after school.*


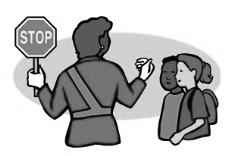
( ) Disagree a lot

( ) Disagree a little

( ) Neither disagree nor agree

( ) Agree a little

( ) Agree a lot

30) There is heavy traffic in the streets where I live.*

( ) Disagree a lot

( ) Disagree a little

( ) Neither disagree nor agree

( ) Agree a little

( ) Agree a lot

31) The roads are safe in my neighbourhood after school.*

( ) Disagree a lot

( ) Disagree a little

( ) Neither disagree nor agree

( ) Agree a little

( ) Agree a lot

32) There are not enough traffic lights and crossings in my neighbourhood after school.*

( ) Disagree a lot

( ) Disagree a little

( ) Neither disagree nor agree

( ) Agree a little

( ) Agree a lot

33) My parents think it is safe for me to be active in the neighbourhood after school.*

( ) Disagree a lot

( ) Disagree a little

( ) Neither disagree nor agree

( ) Agree a little

( ) Agree a lot

Think about when you are at home after school

Please click on the circle which most closely describes **how much you agree or disagree with each statement.**

34) I am scared of dangerous animals in my yard, such as snakes, lizards, dogs or magpies.*

( ) Disagree a lot

( ) Disagree a little

( ) Neither disagree nor agree

( ) Agree a little

( ) Agree a lot

35) There are dangerous objects in my yard, such as rusty scrap metal.*

( ) Disagree a lot

( ) Disagree a little

( ) Neither disagree nor agree

( ) Agree a little

( ) Agree a lot

36) My parents think it is safe for me to play in my yard when I am home alone after school.*

( ) Disagree a lot

( ) Disagree a little

( ) Neither disagree nor agree

( ) Agree a little

( ) Agree a lot

The following statements are about the weather after school

**Please click on the circle which most closely describes how much you agree or disagree with each statement.**

37) When it is raining, it stops me from walking, riding, skating or riding a scooter to and from places after school.*


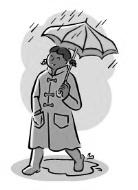
( ) Disagree a lot

( ) Disagree a little

( ) Neither disagree nor agree

( ) Agree a little

( ) Agree a lot

38) When it is too hot, it stops me from walking, riding, skating or riding a scooter to and from places after school.*

( ) Disagree a lot

( ) Disagree a little

( ) Neither disagree nor agree

( ) Agree a little

( ) Agree a lot

39) When it is raining, it stops me from playing actively after school.*

( ) Disagree a lot

( ) Disagree a little

( ) Neither disagree nor agree

( ) Agree a little

( ) Agree a lot

40) When it is too hot, it stops me from playing actively after school.*

( ) Disagree a lot

( ) Disagree a little

( ) Neither disagree nor agree

( ) Agree a little

( ) Agree a lot


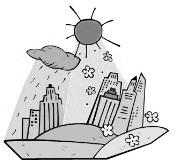


41) When it is raining, it stops me from doing an organised sport or activity after school.*

( ) Disagree a lot

( ) Disagree a little

( ) Neither disagree nor agree

( ) Agree a little

( ) Agree a lot

42) When it is too hot, it stops me from doing an organised sport or activity after school.*

( ) Disagree a lot

( ) Disagree a little

( ) Neither disagree nor agree

( ) Agree a little

( ) Agree a lot

Think about the time you have available after school

Please click on the circle which most closely describes **how much you agree or disagree with each statement.**

43) I have enough time to play actively at home or in my neighbourhood after school.*

( ) Disagree a lot

( ) Disagree a little

( ) Neither disagree nor agree

( ) Agree a little

( ) Agree a lot

44) Homework stops me from playing actively at home or in the neighbourhood after school.*

( ) Disagree a lot


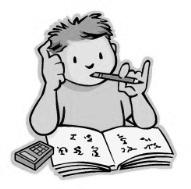
( ) Disagree a little

( ) Neither disagree nor agree

( ) Agree a little

( ) Agree a lot

45) I have enough time to do an organised sport or activity after school.*

( ) Disagree a lot

( ) Disagree a little

( ) Neither disagree nor agree

( ) Agree a little

( ) Agree a lot

46) Homework stops me from doing an organised sport or activity after school.*

( ) Disagree a lot

( ) Disagree a little

( ) Neither disagree nor agree

( ) Agree a little

( ) Agree a lot

The next statements are about the things you have to carry after school

**Please click on the circle which most closely describes how much you agree or disagree with each statement.**


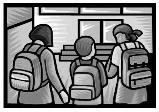
47) My school bag(s) is too heavy for me to walk, ride, skate or scooter home after school.*

( ) Disagree a lot

( ) Disagree a little

( ) Neither disagree nor agree

( ) Agree a little

( ) Agree a lot

48) I don't walk, ride, skate or scooter home from school when I have too many bags to carry.*

( ) Disagree a lot

( ) Disagree a little

( ) Neither disagree nor agree

( ) Agree a little

( ) Agree a lot

Think about what you prefer to do after school

Please click on the circle which most closely describes **how much you agree or disagree with each statement.**

49) I prefer to be active after school instead of watching television or playing electronic games.*


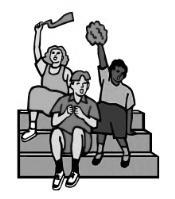
( ) Disagree a lot

( ) Disagree a little

( ) Neither disagree nor agree

( ) Agree a little

( ) Agree a lot

50) I prefer to watch other kids rather than do organised sports and activities after school.*

( ) Disagree a lot

( ) Disagree a little

( ) Neither disagree nor agree

( ) Agree a little

( ) Agree a lot

51) I prefer to do homework rather than be active after school.*

( ) Disagree a lot

( ) Disagree a little

( ) Neither disagree nor agree

( ) Agree a little

( ) Agree a lot

The next section is about your enjoyment of being active after school

**Please click on the circle which most closely describes how much you agree or disagree with each statement.**

52) It is fun being active after school.*

( ) Disagree a lot

( ) Disagree a little


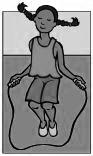
( ) Neither disagree nor agree

( ) Agree a little

( ) Agree a lot

53) Being active after school is the thing I like to do best.*

( ) Disagree a lot

( ) Disagree a little

( ) Neither disagree nor agree

( ) Agree a little

( ) Agree a lot

54) I wish I could do more organised sports or activities after school than I get a chance to.*

( ) Disagree a lot

( ) Disagree a little

( ) Neither disagree nor agree

( ) Agree a little

( ) Agree a lot

55) I don't enjoy doing an organised sport or activity after school.*

( ) Disagree a lot

( ) Disagree a little

( ) Neither disagree nor agree

( ) Agree a little

( ) Agree a lot

56) I don’t feel like playing actively at home or in the neighbourhood after school.*

( ) Disagree a lot

( ) Disagree a little

( ) Neither disagree nor agree

( ) Agree a little

( ) Agree a lot

57) I enjoy being part of an organised sport or activity team.*

( ) Disagree a lot

( ) Disagree a little

( ) Neither disagree nor agree

( ) Agree a little

( ) Agree a lot

58) I don't feel like doing an organised sport or activity after school.*

( ) Disagree a lot

( ) Disagree a little

( ) Neither disagree nor agree

( ) Agree a little

( ) Agree a lot

59) Being active after school is boring.*

( ) Disagree a lot

( ) Disagree a little

( ) Neither disagree nor agree

( ) Agree a little

( ) Agree a lot

60) Being active after school makes me feel good.*

( ) Disagree a lot

( ) Disagree a little

( ) Neither disagree nor agree

( ) Agree a little

( ) Agree a lot

Please click on the circle which most closely describes how much you agree or disagree with each statement.

61) I am too tired to be active after school.*

( ) Disagree a lot

( ) Disagree a little

( ) Neither disagree nor agree

( ) Agree a little

( ) Agree a lot

62) I catch a bus or get driven to and from places after school because I can't be bothered walking or riding.*

( ) Disagree a lot

( ) Disagree a little

( ) Neither disagree nor agree

( ) Agree a little

( ) Agree a lot

63) I walk or ride to and from places after school because I get to hang out with my friends.*

( ) Disagree a lot

( ) Disagree a little

( ) Neither disagree nor agree

( ) Agree a little

( ) Agree a lot

64) I do an organised sport or activity after school because I want to improve my skills.*

( ) Disagree a lot

( ) Disagree a little

( ) Neither disagree nor agree

( ) Agree a little

( ) Agree a lot

65) I do an organised sport or activity after school because I get to hang out with friends.*

( ) Disagree a lot

( ) Disagree a little

( ) Neither disagree nor agree

( ) Agree a little

( ) Agree a lot

66) I do an organised sport or activity after school because I want to meet new people.*

( ) Disagree a lot

( ) Disagree a little

( ) Neither disagree nor agree

( ) Agree a little

( ) Agree a lot

67) I am not active after school because I am scared that I will get injured.*

( ) Disagree a lot

( ) Disagree a little

( ) Neither disagree nor agree

( ) Agree a little

( ) Agree a lot

68) I play in the neighbourhood after school because I get to hang out with my friends.*

( ) Disagree a lot

( ) Disagree a little

( ) Neither disagree nor agree

( ) Agree a little

( ) Agree a lot


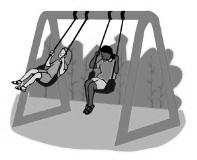


69) I play in the neighbourhood after school because I get to meet new people.*

( ) Disagree a lot

( ) Disagree a little

( ) Neither disagree nor agree

( ) Agree a little

( ) Agree a lot

Still thinking about after school, please click on the circle which most closely describes how much you agree or disagree with each statement.

70) I ride, walk, skate or scooter to and from places after school because it gets me fit.*

( ) Disagree a lot


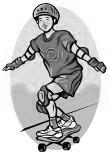
( ) Disagree a little

( ) Neither disagree nor agree

( ) Agree a little

( ) Agree a lot

71) Playing outside after school keeps me healthy.*

( ) Disagree a lot

( ) Disagree a little

( ) Neither disagree nor agree

( ) Agree a little

( ) Agree a lot

72) I do an organised sport or activity after school because I have nothing else to do.*

( ) Disagree a lot

( ) Disagree a little

( ) Neither disagree nor agree

( ) Agree a little

( ) Agree a lot

73) I don't participate in some activities after school because it is only a boys/girls activity.*

( ) Disagree a lot

( ) Disagree a little

( ) Neither disagree nor agree

( ) Agree a little

( ) Agree a lot

74) I play actively at home or in the neighbourhood after school because it gives me something to do.*

( ) Disagree a lot

( ) Disagree a little

( ) Neither disagree nor agree

( ) Agree a little

( ) Agree a lot

75) I do an organised sport or activity after school because it gets me fit.*

( ) Disagree a lot

( ) Disagree a little

( ) Neither disagree nor agree

( ) Agree a little

( ) Agree a lot

76) I am active after school because I don't want to put on weight.*

( ) Disagree a lot

( ) Disagree a little

( ) Neither disagree nor agree

( ) Agree a little

( ) Agree a lot

The following statements are about your confidence to be active after school

**Please click on the circle which most closely describes how much you agree or disagree with each statement.**

77) I am confident that I can ask my parent or another adult to take me to an organised sport or activity after school.*

( ) Disagree a lot

( ) Disagree a little

( ) Neither disagree nor agree

( ) Agree a little

( ) Agree a lot

78) I am confident that I can ask my parent or another adult to take me somewhere I can play actively after school.*

( ) Disagree a lot

( ) Disagree a little

( ) Neither disagree nor agree

( ) Agree a little


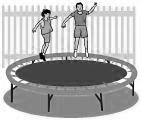
( ) Agree a lot

79) I am confident that I can be active after school on most days.*

( ) Disagree a lot

( ) Disagree a little

( ) Neither disagree nor agree

( ) Agree a little

( ) Agree a lot

80) I am confident that I can be active after school on most days even if I could watch TV or play video games instead.*

( ) Disagree a lot

( ) Disagree a little

( ) Neither disagree nor agree

( ) Agree a little

( ) Agree a lot

81) I am confident that I can be active after school on most days even if it is hot or cold outside.*

( ) Disagree a lot

( ) Disagree a little

( ) Neither disagree nor agree

( ) Agree a little

( ) Agree a lot

82) I am confident that I can ask friends to be active with me after school on most days.*

( ) Disagree a lot

( ) Disagree a little

( ) Neither disagree nor agree

( ) Agree a little

( ) Agree a lot

83) I am confident that I can be active after school on most days even if I have to stay at home.*

( ) Disagree a lot

( ) Disagree a little

( ) Neither disagree nor agree

( ) Agree a little

( ) Agree a lot

84) I am confident that I can be active after school on most days even if I am being bullied.*

( ) Disagree a lot

( ) Disagree a little

( ) Neither disagree nor agree

( ) Agree a little

( ) Agree a lot

85) I am confident that I can be active after school on most days no matter how busy I am.*

( ) Disagree a lot

( ) Disagree a little

( ) Neither disagree nor agree

( ) Agree a little

( ) Agree a lot

The next statements are about how good you think you are at doing activities after school

Please click on the circle which most closely describes **how much you agree or disagree with each statement.**

86) I ride my bike, skateboard or scooter to and from places after school because I think I am good at it.*

( ) Disagree a lot


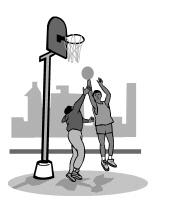
( ) Disagree a little

( ) Neither disagree nor agree

( ) Agree a little

( ) Agree a lot

87) I play active games after school because I think I am good at them.*

( ) Disagree a lot

( ) Disagree a little

( ) Neither disagree nor agree

( ) Agree a little

( ) Agree a lot

88) I do an organised sport or activity after school because I think I am good at it.*

( ) Disagree a lot

( ) Disagree a little

( ) Neither disagree nor agree

( ) Agree a little

( ) Agree a lot

89) It is not worth doing an organised sport or activity after school because I am not good at it.*

( ) Disagree a lot

( ) Disagree a little

( ) Neither disagree nor agree

( ) Agree a little

( ) Agree a lot

90) I don't do an organised sport or activity after school because other kids are better than me.*

( ) Disagree a lot

( ) Disagree a little

( ) Neither disagree nor agree

( ) Agree a little

( ) Agree a lot

Now think about your friends and the people around you after school

Please click on the circle which most closely describes **how much you agree or disagree with each statement.**

91) Bullying stops me from being active after school.*

( ) Disagree a lot

( ) Disagree a little

( ) Neither disagree nor agree

( ) Agree a little

( ) Agree a lot

92) I am not active after school because I have no one to play with.*

( ) Disagree a lot

( ) Disagree a little

( ) Neither disagree nor agree

( ) Agree a little

( ) Agree a lot

93) My friends encourage me to be active after school.*

( ) Disagree a lot

( ) Disagree a little

( ) Neither disagree nor agree

( ) Agree a little

( ) Agree a lot

94)

I walk, ride, skate or scooter to and from places with friends after school.*


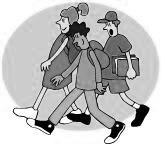
( ) Disagree a lot

( ) Disagree a little

( ) Neither disagree nor agree

( ) Agree a little

( ) Agree a lot

95) My friends tell me I am doing well at sport.*

( ) Disagree a lot

( ) Disagree a little

( ) Neither disagree nor agree

( ) Agree a little

( ) Agree a lot

96) I play with friends in the neighbourhood after school.*

( ) Disagree a lot

( ) Disagree a little

( ) Neither disagree nor agree

( ) Agree a little

( ) Agree a lot

97) I do an organised sport or activity with friends after school.*

( ) Disagree a lot

( ) Disagree a little

( ) Neither disagree nor agree

( ) Agree a little

( ) Agree a lot

Now think about your parents and your family

Please click on the circle which most closely describes **how much you agree or disagree with each statement.**


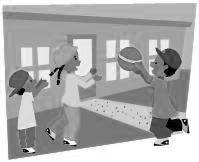
98) I have brothers/sisters who play actively with me after school.*

( ) Disagree a lot

( ) Disagree a little

( ) Neither disagree nor agree

( ) Agree a little

( ) Agree a lot

99) My parents play actively with me after school.*

( ) Disagree a lot

( ) Disagree a little

( ) Neither disagree nor agree

( ) Agree a little

( ) Agree a lot

100) My parents encourage me to play outside after school.*

( ) Disagree a lot

( ) Disagree a little

( ) Neither disagree nor agree

( ) Agree a little

( ) Agree a lot

101) My parents are not home after school to supervise my play.*

( ) Disagree a lot

( ) Disagree a little

( ) Neither disagree nor agree

( ) Agree a little

( ) Agree a lot

102) My parents make me go outside and play after school.*

( ) Disagree a lot

( ) Disagree a little

( ) Neither disagree nor agree

( ) Agree a little

( ) Agree a lot

103) My parents are too busy to play with me after school.*

( ) Disagree a lot

( ) Disagree a little

( ) Neither disagree nor agree

( ) Agree a little

( ) Agree a lot

104) I always have to tell my parents where I am when I go out after school.*

( ) Disagree a lot

( ) Disagree a little

( ) Neither disagree nor agree

( ) Agree a little

( ) Agree a lot

105) My parents make me help around the house, which stops me from being active after school.*

( ) Disagree a lot

( ) Disagree a little

( ) Neither disagree nor agree

( ) Agree a little

( ) Agree a lot

106) If I am going out after school, I always have to be back by a certain time.*

( ) Disagree a lot

( ) Disagree a little

( ) Neither disagree nor agree

( ) Agree a little

( ) Agree a lot

107) My parents won't let me ride, walk, skate or scooter to and from places after school.*

( ) Disagree a lot

( ) Disagree a little

( ) Neither disagree nor agree

( ) Agree a little

( ) Agree a lot

108) My family always watch me do an organised sport or activity after school.*

( ) Disagree a lot

( ) Disagree a little

( ) Neither disagree nor agree

( ) Agree a little

( ) Agree a lot

109) My parents help me practise sport after school.*

( ) Disagree a lot

( ) Disagree a little

( ) Neither disagree nor agree

( ) Agree a little

( ) Agree a lot

110) I don't do an organised sport or activity after school because my parents work late.*

( ) Disagree a lot

( ) Disagree a little

( ) Neither disagree nor agree

( ) Agree a little

( ) Agree a lot

111) My parents won't let me do an organised sport or activity because I am already doing too many other activities.*


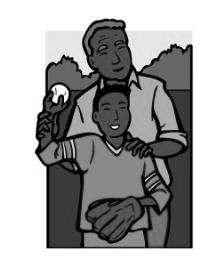
( ) Disagree a lot

( ) Disagree a little

( ) Neither disagree nor agree

( ) Agree a little

( ) Agree a lot

112) My parents encourage me to do an organised sport or activity after school.*

( ) Disagree a lot

( ) Disagree a little

( ) Neither disagree nor agree

( ) Agree a little

( ) Agree a lot

113) My family tell me I am doing well at my after school organised sport or activity.*

( ) Disagree a lot

( ) Disagree a little

( ) Neither disagree nor agree

( ) Agree a little

( ) Agree a lot

114) We have a rule at home that we have to do an organised sport or activity after school.*

( ) Disagree a lot

( ) Disagree a little

( ) Neither disagree nor agree

( ) Agree a little

( ) Agree a lot

115) I am not allowed to do an organised sport or activity after school because my parents are scared that I might get hurt.*

( ) Disagree a lot

( ) Disagree a little


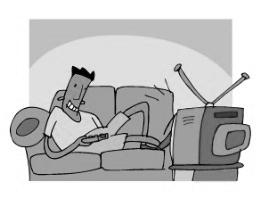
( ) Neither disagree nor agree

( ) Agree a little

( ) Agree a lot

116) My parents have rules about watching TV and playing electronic games after school.*

( ) Disagree a lot

( ) Disagree a little

( ) Neither disagree nor agree

( ) Agree a little

( ) Agree a lot

What organised sports and activities have you done in the last year?

117) What ORGANISED SPORTS AND ACTIVITIES have you done in the LAST YEAR?

**Example:**

|  | **Activity** | **Time of year** | | **Who do you do this sport or activity for?** | | |
| --- | --- | --- | --- | --- | --- | --- |
|  |  | **Summer** | **Winter** | **School** | **Club** | **Other** |
| 1 |  | [ ] | [ ] | [ ] | [ ] | [ ] |
| 2 |  | [ ] | [ ] | [ ] | [ ] | [ ] |
| 3 |  | [ ] | [ ] | [ ] | [ ] | [ ] |
| 4 |  | [ ] | [ ] | [ ] | [ ] | [ ] |
| 5 |  | [ ] | [ ] | [ ] | [ ] | [ ] |
| 6 |  | [ ] | [ ] | [ ] | [ ] | [ ] |

This statement is about your coordination

Please click on the circle which most closely describes **how much you agree or disagree with each statement.**

118) I am just as coordinated as kids of my age and gender.*

( ) Disagree a lot

( ) Disagree a little

( ) Neither disagree nor agree

( ) Agree a little

( ) Agree a lot

This statement is about how you view your body

Please click on the circle which most closely describes **how much you agree or disagree with each statement.**

119) I am shy about how my body looks.*

( ) Disagree a lot

( ) Disagree a little

( ) Neither disagree nor agree

( ) Agree a little

( ) Agree a lot

The next statement is about how much you enjoy PE lessons

Please click on the circle which most closely describes **how much you agree or disagree with each statement.**

120) I really like doing PE at school.*

( ) Disagree a lot

( ) Disagree a little

( ) Neither disagree nor agree

( ) Agree a little

( ) Agree a lot

Thank You!

Thank you for completing this survey.
